# An integrin-based quercetin 7-rhamnoside liver-targeted delivery liposomes for intrahepatic cholestasis in pregnancy

**DOI:** 10.1016/j.mtbio.2025.102031

**Published:** 2025-06-27

**Authors:** Xiaoying Feng, Ling Dai, Yanfang Guo, Liuting Zhong, Yuxiu Zheng, Senling Feng, Liping Cao, Zhongwen Yuan

**Affiliations:** a Department of Pharmacy, Guangdong Provincial Key Laboratory of Major Obstetric Diseases, Guangdong Provincial Clinical Research Center for Obstetrics and Gynecology, The Third Affiliated Hospital, Guangzhou Medical University, Guangzhou, 510150, China; b School of Pharmaceutical Sciences, Guangzhou Medical University, Guangzhou, 511436, China; c Department of Pharmacy, Shenzhen Bao'an Chinese Medicine Hospital, Guangzhou University of Chinese Medicine, Shenzhen, 518100, China

**Keywords:** Intrahepatic cholestasis in pregnancy, Quercetin 7-rhamnoside, Mitochondrial function, Bile acid metabolism, Peptide A20FMDV2, Nanodrug delivery system

## Abstract

Intrahepatic cholestasis in pregnancy (ICP) is a characteristic disease during the perinatal period; however, its therapy remains unsatisfactory, and the pathogenesis remains unclear. The ameliorative effect of naturally occurring quercetin 7-rhamnoside (Q7R) in cholestasis has been established. In this study, we aimed to establish a nanoparticle-based peptide, A20FMDV2-modified liposome (t-QL), to encapsulate and deliver Q7R. Q7R bioavailability improved significantly when liposomes were used as carriers. This peptide A20FMDV2-modified nanosystem targeted integrin αvβ6 on biliary epithelial cells and improved stillbirth rates and liver function indicators better than free Q7R without a carrier. Q7R improved ICP by regulating mitochondrial function and bile metabolism. Our nanosystem provides a promising nanotherapeutic strategy for applying Q7R in ICP. We also elucidated a therapeutic mechanism underlying the action of ICP by simultaneously targeting mitochondrial structure and function, as well as bile acid metabolism.

## Graphical abstract

Schematic diagram illustrating the preparation of t-QL and their therapeutic mechanisms for treating ICP.

**Figure:**
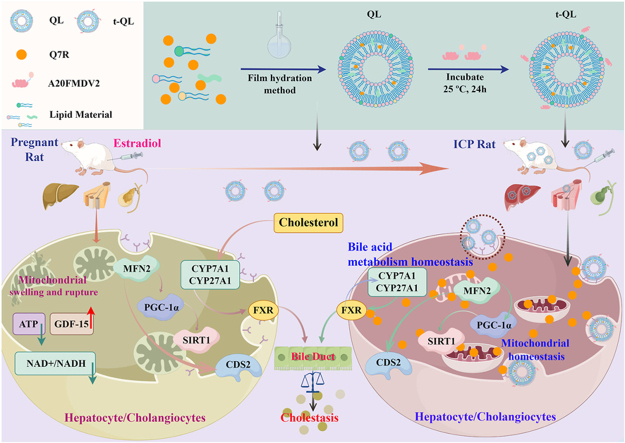

## 1 Introduction

Intrahepatic cholestasis of pregnancy (ICP) is a pregnancy-specific liver disease characterised by pruritus, elevated serum bile acids (BAs), serum total BA concentrations exceeding 10 μmol/L, and abnormal liver function, resulting in various adverse pregnancy outcomes including intrauterine foetal demise. However, the pathogenesis of ICP remains unclear [1]. Current research indicates that the *SLC10A1 p. Ser267* Phe variant and related polymorphisms at the 14q24.1 locus confer a 16.56-fold elevated risk of ICP, providing novel insights into the genetic susceptibility to ICP in East Asian populations [2]. Genetic defects in BA synthesis and transport–involving key genes such as ATP binding cassette subfamily B member 4 (ABCB4), ATP binding cassette transporter C3 (ABCC3), cholesterol 7a-hydroxylase (CYP7A1), and sterol 27-hydroxylase (CYP27A1)–have been mechanistically linked to ICP pathogenesis. Furthermore, immune-related genes, including chemokine (C-C motif) ligand 6, chemokine (C-C motif) ligand 14, and the chemokine (C-C motif) ligand 1 – C-X-C motif chemokine receptor 2 axis, may contribute to disease development through dysregulated inflammatory responses [3,4]. Additionally, ICP has been associated with decreased endogenous levels of selenium and vitamin D, as well as elevated maternal triglyceride concentrations during the second and third trimesters [5,6]. Although the precise molecular mechanisms underlying ICP remain incompletely defined, the estrogen-bile acid axis continues to be the primary focus of current research [7]. The liver is the primary site of oestrogen metabolism, and liver diseases are usually associated with abnormal oestrogen levels. Studies have confirmed that oxidative stress and mitochondrial dysfunction are the significantly contribute to the pathogenesis of hepatic cholestasis. Cholestasis causes mitochondrial dysfunction and oxidative injury. The mitochondria control cellular energy metabolism, hormones, BA synthesis via biogenesis, fusion, fission, and mitophagy [8,9]. Deoxycholic acid (DCA) and cholic acid (CA) cause a decreased mitochondrial mass and decreased mitochondrial biogenesis [10]. The liver mitochondria in cholestatic mice exhibited collapsed mitochondrial membrane potential, mitochondrial swelling, increased reactive oxygen species (ROS), and lipid peroxidation [11]. Alterations in the mitochondrial redox state comprise a significant characteristic of liver injury in cholestatic animal models [12]. ICP in patients and mice can be complicated by elevated oxidative stress and decreased mitochondrial matrix PRDX3 expressions in the placenta. Chenodeoxycholic acid (CDCA) or DCA under starvation significantly decreases the mitochondrial membrane potential and intracellular adenosine triphosphate (ATP) contents in HTR8 cells [13]. Therefore, the role of the mitochondria in the pathological process of ICP requires further validation.

Ursodeoxycholic acid (UDCA, 10–15 mg/kg/day) is the recommended first-choice drug for treating ICP, and S-adenosyl-methionine (SAM, 450–1000 mg/day) is used as a second-line drug or combination therapy for ICP treatment. However, the roles of UDCA and SAM remain controversial. Therefore, developing new, safe, and effective therapeutic drugs is essential [14,15]. Quercetin 7-rhamnoside (Q7R) is a major active component of *Hypericum japonicum*. Previous research has suggested that Q7R exerts hepatoprotective effects by regulating BA secretion, alleviating inflammation, and antioxidation. Q7R is a promising ICP medication [16]; however, its water solubility is excessively poor, restricting its clinical use. Therefore, designing a suitable delivery system is crucial to expanding the Q7R application.

A liposome is a well-known nanoscale vesicle drug and mRNA delivery carrier with many advantages, including non-immunogenicity, superior biodegradability, and controllable release kinetics. Liposomes carry drugs with different polarities in their phospholipid bilayer and inner core, improving bioavailability and organ targeting. These advanced biological properties have prompted the common application of liposome-based drug delivery systems in pharmaceutical fields involving antitumour, immune regulation, and anti-inflammatory activities [17,18]. Modifying the surface of the liposome with special ligand content of peptide, chitosan, lactoferrin, and sialic acid improves the targeting efficiency and release kinetics of liposomes [[19], [20], [21]].

ICP damages bile duct and liver cells, inducing ductular reaction and triggering the proliferation of biliary epithelial cells (BEC) and interlobular bile ducts [22,23]. Integrin ανβ6 is a cell surface receptor belonging to the integrin family and is rapidly upregulated on cells of epithelial lineage during in BEC injury and cholestasis model [24]. Chronic injury to intrahepatic bile duct epithelial cells elicits over-expression of integrin αvβ6 and drives fibrogenesis via adhesion to fibronectin and auto/paracrine TGFβ1 activation [25,26]. Consequently, integrin αvβ6 emerges as a promising therapeutic target for mitigating cholestatic injury and hepatic fibrosis progression. ICP exhibits fundamental pathophysiological hallmarks of cholestatic liver injury. Targeting integrin αvβ6 for ICP therapy is grounded in robust mechanistic rationale [[26], [27], [28]].

A20FMDV2 is a 20-mer peptide derived from the foot-and-mouth disease virus. This peptide exhibits a high affinity for integrin αVβ6. Peptide A20FMDV2 has been applied to modify various therapeutic drugs and diagnostic reagent delivery systems based on its unique targeting affinity [29,30].

Targeted modification of the surface of liposomes using peptide A20FMDV2 promotes targeted accumulation of drugs at the lesion site and improves therapeutic efficacy, providing novel and promising strategies for the effective treatment of ICP. Here, we studied the beneficial effects of Q7R therapy in an ICP rat model. Our data suggest that tail vein injection pre-administration of the peptide A20FMDV2 -modified liposome encapsulated Q7R resulted in alleviating the clinical symptoms of ICP. In addition, typical mitochondrial function and bile acid metabolism signalling pathway proteins were studied to understand the mechanism of ICP alleviation with Q7R administration. A schematic diagram illustrating the preparation process of peptide-modified targeting liposomes and their therapeutic mechanism for ICP is shown in Graphical abstract (Draw by Figdraw).

## 2 Materials and methods

### 2.1 Chemicals and reagents

Quercetin 7-rhamnoside (Q7R, 99.32 %) was purchased from Biopurify (Chengdu Bio-Purified Plant Chemicals Co., Ltd., Chengdu, China). Hydrophospholipids (HSPC-95A, ≥ 95.0 %) and Soy Lecithin 98 (SPC, 98 %) were purchased from Avito (Shanghai Avito Biotechnology Co., Ltd., Shanghai, China). Sodium cholesterol sulphate (SCS) was purchased from Yuanye (Shanghai Yuanye Biotechnology Co., Ltd., China). The LavaPep Peptide Quantification kit (LP-022010, Lot No. L01323) was purchased from Gel Company (Gel Company, Inc., San Francisco, CA, USA). The cysteine-modified A20FMDV2 peptide (Sequence: NAVPNLRGDLQVLAQKVARTC; HPLC purity, 96.9 %; theoretical molecular weight: 2266.63) was synthesised by GenScript (GenScript Biotech Corporation, Nanjing, China). All other solvents and reagents used were of analytical grade.

### 2.2 Cell lines and animals

Hepa 1–6 and HCCC-9810 cells were purchased from Pricella Biotechnology (Wuhan, China) and cultured in DMEM (Hyclone, Beijing, China) or RPMI-1640 media in a 37 °C, 5 % CO_2_, saturated humidity incubator. All culture media contained 10 % foetal bovine serum (FBS, Hyclone, Beijing, China) and 1 % penicillin-streptomycin.

Sprague–Dawley (SD) rats (180–220 g; medical experimental animal number, SCXK (YUE) 2021-0059; animal certification number, 44827200005836) and Kunming mice (18–20 g; animal certification number, 44827200008048; medical experimental animal number, SCXK (YUE) 2023-0059) were provided by Guangzhou Ruige Biotechnology Co., Ltd. New Zealand rabbits (2.4–2.7 kg; animal certification number, 44411600013759; medical experimental animal number, SCXK (YUE) 2019-0035) were provided by the Medical Experimental Animal Centre of Guangdong Province.

All animals were allowed to acclimate to the housing conditions for seven days prior to experimentation. The animals were housed in a temperature- (22 °C) and humidity-controlled (70 %) room with a 12 h dark-light cycle, and fed a diet of standard pellets and water. All animals were maintained with a treatment protocol for this study, which was approved by Guangzhou Medical University. Furthermore, the animals used in this study were treated humanely and in compliance with institutional animal care guidelines. The experimental protocols were approved by the Ethics Committee of The Third Affiliated Hospital of Guangzhou Medical University (Ethical approval: YILUNHUISHEN [2024] # 138).

### 2.3 Preparation of Q7R liposomes

Single factor and Box-Behnken response surface design were conducted to optimise the prescription and preparation process of Q7R liposomes; the experimental results are presented in Supplementary materials 2 and 3. After optimisation, untargeted Q7R liposome (QL) was prepared viathin-film hydration and sonication. The oil phase comprised 9.83, 136.10, 43.90, 28.89, and 12 mg of Q7R, SPC, HSPC, SCS, and DSPE-PEG2000, respectively; in the oil phase, 60 mL of methanol was added and dissolved using an ultrasonic cleaner (SK5210HP, Shanghai, China). The above solution was placed in a round-bottom flask (1000 mL) and a thin lipid film was generated by evaporating the solvent under reduced pressure with a rotary evaporator (Rotavapor® RE-201D, Zhengzhou, China) at 55 °C. The film was hydrated by adding 15 mL of phosphate-bufferedsolution (PBS). Subsequently, probe sonication (250 W, JY92-II, Ningbo, China) was conducted for 240 s with an ice-water bath. The unencapsulated Q7R was eliminated via filtration through a 0.22-μm microporous filter membrane, and the resulting liposome solution was stored at 4 °C.

### 2.4 Preparation of targeted Q7R liposomes (t-QL) and peptide quantification

To increase the hepatic and bile duct targeting ability of QL, the liposome surface was modified with peptide A20FMDV2. Liposomes were formed as described above, with 12 mg of DSPE-mPEG2000-Maleimide (DSPE-PEG2000-Mal) replacing DSPE-PEG2000. The liposome was incubated with the A20FMDV2 peptide (Figs. S1 and 2) overnight at 25 °C (10 μg peptide/mg lipid); the thiol group of the C-terminal cysteine residue of the peptide reacted with the maleimide group on the DSPE-PEG2000-Mal to form a thioether bond. The optimal peptide content is discussed in Supplementary material 3. Excess peptides were removed through overnight dialysis against PBS using dialysis membranes with a molecular weight cutoff of 12,000–14,000 kDa at 4 °C. The A20FMDV2 peptide was quantified using a LavaPep™ Fluorescent Peptide and Protein Quantification Kit according to the manufacturer's instructions. For optimal peptide content of liposomes and cellular uptake studies, fluorescent liposomes were formed using coumarin 6 (C6, Aladdin, Shanghai, China), replacing Q7R. For fluorescence imaging of *in vivo* distribution in mice, fluorescent liposomes were formed using DiR iodide (DiR, > 95 %, Bergolin, Dalian, China) replacing Q7R.

### 2.5 Characterisation of liposomes

The mean particle size, Zeta potential, and polydispersity index (PDI) of liposomes were analysed through dynamic laser scattering using a Malvern Zetasizer Nano ZS instrument at 25 °C (ZATA SIZER1000H, Malvern, UK) with three repetitions. The PDI was also obtained via the cumulative volume analysis of the correlation function. Liposomes were studied morphologically using a transmission electron microscope (TEM, Talos L120c, Thermo Fisher, Waltham, MA, USA).

### 2.6 Measurement of the entrapment efficiency (EE %) of Q7R liposomes

To determine the EE% of Q7R liposomes, QL and t-QL were collected through microporous membrane filtration (0.22 μm) and centrifugation (15,000 × *g* for 15 min at 4 °C). Subsequently, 200 μL of the upper liposome solution was mixed with 200 μL of N, N-dimethylformamide (DMF) and 600 μL of methanol for demulsification, followed by centrifugation at 15,000 × *g* and 4 °C for 15 min. Next, the supernatant was injected into an Agilent 1260 high-performance liquid chromatography (HPLC) system for Q7R analysis. The detection method, methodological validation, and results are shown in Supplementary materials 4.

The Q7R content was calculated using an external standard method, and the EE % was calculated using the following equation:

$$\text{EE}\;\left(\%\right)={\text{Mass}}_{\text{encapsulate}}/{\text{Mass}}_{\text{total}}\times 100\%$$

where Mass _total_ is the analysed amount of total Q7R, and Mass _encapsulate_ is the analysed amount of Q7R in the filtered liposome solution.

### 2.7 In vitro liposomestability

The stability of QL and t-QL was assessed by determining the drug amount *in vitro*. Q7R-loaded liposomes were added to PBS or 50 % plasma (1:1, *v/v*) and incubated at 37 °C with gentle shaking. The drug content was measured at 0, 1, 2, 4, 6, 8, 12, and 24 h using the method described in Section 2.6.

### 2.8 In vitro release study

*In vitro* drug release studies of Q7R-liposomes were conducted using dynamic dialysis. Q7R-liposome was added to an activated dialysis bag (12,000–14,000 kDa) and sealed up. The sealed dialysis bag was placed in 100 mL of PBS for stirring dialysis (containing 2 % Tween-80) at 37 ± 0.5 °C and 100 × *g*. At the specified sampling time points (0, 1, 2, 4, 6, 8, 10, 12, 24, 36, and 48 h), 1 mL of sample solution was aspirated, and an equal volume of fresh dialysate was added. The sample was concentrated, dried under vacuum, and reconstituted usingan ultrapure water-DMF-methanol (2:2:8) mixed solution. The Q7R contents at different time points were determined using a Shimadzu HPLC tandem AB Sciex 3200 MD mass spectrometer (LC-MS/MS), and the drug concentration at each time point was evaluated using a standard curve to fit the *in vitro* release curve. The detailed detection method is described in Supplementary material 4.

### 2.9 Haemolysis test

The red blood cells (RBCs) of fresh blood samples of New Zealand rabbits were isolated via centrifugation at 3,000 *× g* and 4 °C for 10 min, washed thrice in saline, and a 0.25 % (*v/v)* solution of RBCs was prepared in saline. QL and t-QL solutions were diluted to the predetermined concentration, and an equal volume of QL and t-QL solutions was added to the 0.25 mL of the RBC sample (0.25 %, *v/v*) and gently mixed to achieve an equivalent final Q7R concentration (0.05, 0.1, 0.2, 0.5, 1, 2, 5, 10, 20, 50, 100, and 200 μg/mL). Equal volumes of saline and 1 % Triton-X100 were used as the negative and positive controls, respectively. The above samples were incubated at 37 °C for 2 h and subsequently centrifuged at 3,000 *× g* for 10 min, and 100 μL of the supernatant was extracted into a 96-well plate. The absorbance was measured at 540 nm, and the haemolysis percent was calculated.

$$\text{Haemolysis}\%=\left({\text{Absorbance}}_{\text{treated}}-{\text{Absorbance}}_{\text{negative}\;\text{control}}\right)/\left({\text{Absorbance}}_{\text{positive}\;\text{control}}-{\text{Absorbance}}_{\text{negative}\;\text{control}}\right)\times 100\%\text{.}$$

### 2.10 Evaluation of cellular uptake and *in vivo* liver targeting

To assess the liver targeting efficiency of the modified liposomes, its uptake characteristics in the cell model were observed through laser confocal scanning microscopy (LSCM). Hepa1-6 and HCCC-9810 cells were inoculated in confocal dishes (5 × 10^4^ cells/well). After 24 h of incubation, the original medium was replaced with a fresh medium containing C6 solution, C6-Lip, and t-C6-Lip (C6, 10 μg/mL) and incubated for 1, 2, and 4 h. After incubation, the medium was removed and the cells fixed with 4 % paraformaldehyde for 20 min. The cells were stained with 4′,6-diamidino-2-phenylindole (DAPI at 2.5 μg/mL) and washed thrice using sterile PBS. A Nikon LSCM was used to observe the cellular uptake and fluorescence distribution of DAPI and C6.

To assess the biodistribution and hepatic targeting efficiency of the liposomal delivery system, mice were injected with 8 μg/kg of t-DiR Lip, DiR Lip, and free DiR solution in the tail vein. The fluorescence of mouse liver was observed with an *in vivo* imaging system at 1, 5, 8, and 24 h after tail vein injection. Subsequently, 24 h after *in vivo* imaging, the mice were euthanised and the liver tissues were collected to observe the fluorescence using an *in vivo* imaging system.

### 2.11 Pharmacodynamic evaluation of t-QL

Forty-eight female rats (8 weeks) in oestrus were housed in cages with fertile males at a 3:1 ratio between males and females. The female rats were tested for pregnancy using the vaginal smear, and the presence of spermatozoa indicated Day 1 of pregnancy. On the 10th day of gestation, the female SD rats were randomly divided into eight groups (*n* = 6 rats per group), Based on our preliminary studies and the relevant literature, we selected the administered dose of Q7R.

1) Control and 2) model: blank solvent, intravenous injections (*i.v.*);
3) Positive control: S-adenosyl-L-methionine-1,4-butane-disulfonate (SAM, H20143203, Lot number: 230112, Zhejiang Zhenyuan Pharmaceutical Co., Ltd.) (90 mg/kg (*i.m.*));
4) Q7R: Q7R solution (5 mg/kg, *i.v.*);
5) QL group: QL (5 mg/kg, *i.v.*);
6) t-QL low-dose: t-QL (t-QL-L, 2.5 mg/kg, *i.v.*);
7) t-QL medium-dose: t-QL (t-QL-M, 5 mg/kg, *i.v.*);
8) t-QL high-dose: t-QL (t-QL-H, 10 mg/kg, *i.v.*).

The drugs were administered once daily for 10 consecutive days. In addition, an ICP animal model was established using 2.5 mg/kg (*i.m.*) of estradiol benzoate injection (EB, 120032511, Lot number: 20240102, Hefei Xinkexin Animal Medicine Co., Ltd.) once daily for 1 week from Day 12 of gestation [31]. Normal control rats were injected with an equal volume of saline during the same period. The model drug was administered 1 h before injecting the treatment drug.

For all pregnant rats in each group, pregnancy was terminated when bloody vaginal discharge was observed, and the body weights of the rats were recorded. Subsequently, the rats were anesthetised and euthanised with sodium pentobarbital (60 mg/kg). The blood sample was collected from the abdominal aorta, and the serum was separated. Liver was weighed, under went quick freezing with liquid nitrogen, and was stored at −80 °C. The number of embryonic deaths and embryonic survivors were recorded, and the liver index and embryonic lethality were calculated. The equations were as follows.

(1) Liver to body weight ratio = Liver weight/Rat body weight × 100 %;
(2) Embryonic lethality = Number of embryo deaths/Total number of embryos × 100 %.

### 2.12 Histopathology and biochemical analysis

Fresh rat livers were isolated and fixed in 4 % paraformaldehyde, then embedded in paraffin and sliced. Slices were subjected to haematoxylin and eosin (H&E) and Masson's staining. Histological changes were evaluated by randomly selecting visual fields using an upright microscope (Nikon, Japan). The serumor liver levels of alkaline phosphatase (AKP), alanine transaminase (ALT), aspartate transaminase (AST), gamma-glutamyl transferase (γ-GGT), total bilirubin (TBIL), total BA (TBA), superoxide dismutase (SOD), malondialdehyde (MDA), NAD^+^/NADH ratio, adenosine triphosphate (ATP) and growth differentiation factor 15 (GDF-15) were evaluated using commercial kits according to the manufacturers' instructions.

### 2.13 Evaluation of hepatocyte mitochondrial morphology

Isolated fresh rat livers (approximately 2 × 2 × 2 mm) were rapidly fixed and embedded with an electron microscope fixative. Sections were stained using dicumyl acetate and lead citrate. The mitochondria were evaluated using a Tecnai G2 Spirit transmission electron microscope (FEI Czech Republic s.r.o., Jihomoravsky, Czech Republic) with randomly selected fields of view.

### 2.14 RT-PCR analysis

Total RNA was isolated from the frozen liver of rat using TRIzol® reagent (Life Technology, NY, USA). RNA was purified and reverse-transcribed using SweScript All-in-One RT SuperMix for qPCR Kit with gDNA Remover (Servicebio, Wuhan, China). Quantitative real-time PCR was performed using Universal Blue SYBR Green qPCR Master Mix (Servicebio, Wuhan, China) on a CFX Connect Real-Time PCR System (Bio-Rad, Hercules, CA, USA). Fold change of gene expression levels was calculated using cycle threshold (Ct) values and normalised to *GAPDH*. The relative quantities of mRNA expression were determined using the comparative CT (ΔΔCT) method. The primer sequences are shown in Table S9.

### 2.15 Western blotting

The proteins from the liver tissues of rats were extracted and quantified using the bicinchoninic acid method. The liver protein underwent electrophoresis, followed by membrane transfer. After blocking, the membranes were incubated with anti-FXR, anti-CYP27A1, anti-CDS2, and anti-MFN2 antibodies. The membranes were washed thrice with TBST and then incubated for 1 h with horseradish peroxidase-labelled secondary antibodies at a dilution of 1:3000. After washing thrice using TBST, protein detection was conducted using an efficient chemiluminescence reagent under Gel Imaging System (Bio-Rad, Hercules, CA, USA). ImageJ software was used for subsequent semiquantitative analysis. The antibody information is listed in Supplementary material 7 (Table S10).

### 2.16 Analysis of serum BAs profile

Serum (100 μL) was added to 500 μL of cold methanol and 10 μL of mixed internal standard (200 ng/mL), followed by vortexing for 1 min.The precipitated protein was centrifuged (5804R, Eppendorf, Hamburg, Germany) at 14,000 × *g* and 4 °C for 20 min. The supernatant was removed and freeze-dried, and the samples were stored at −80 °C for use. Agilent 1290 Infinity UPLC/AB Sciex 5500 Qtrap Mass Spectrometer was used for BA profile analysis. The detailed detection method is described in Supplementary material 8.

### 2.17 Statistical analysis

Data are expressed as mean ± standard deviation. Statistical analyses including a two-tailed Student's t-tests and one-way analysis of variance (ANOVA) were conducted using GraphPad Prism 9.0 (GraphPad Software Inc., CA, USA), while normality testing and Pearson's correlation analysis were performed using SPSS 26.0 (IBM, Inc., Armonk, NY, USA). Principal component analysis (PCA) was conducted with SIMCA 14.1 (Umetrics AB, Umea, Sweden). Heatmap analysis was performed using MetaboAnalyst 5.0 (https://www.metaboanalyst.ca/faces/home.xhtml, Xia Lab @ McGill). *p* *<* *0.05* was considered statistically significant.

## 3 Results and discussion

### 3.1 Optimisation of the liposome formula and preparation process

Single factor investigation involved the SPC/HSPC ratio, TPC/SCS ratio, TPC/Q7R ratio, ultrasonic power, and ultrasonic time to evaluate the key influences on Q7R liposome preparation. Single factor investigation indicated that the SPC/HSPC, TPC/SCS, and TPC/Q7R ratios were the main influencing factors (Figs. S3 and S4).

**Fig. 1:**
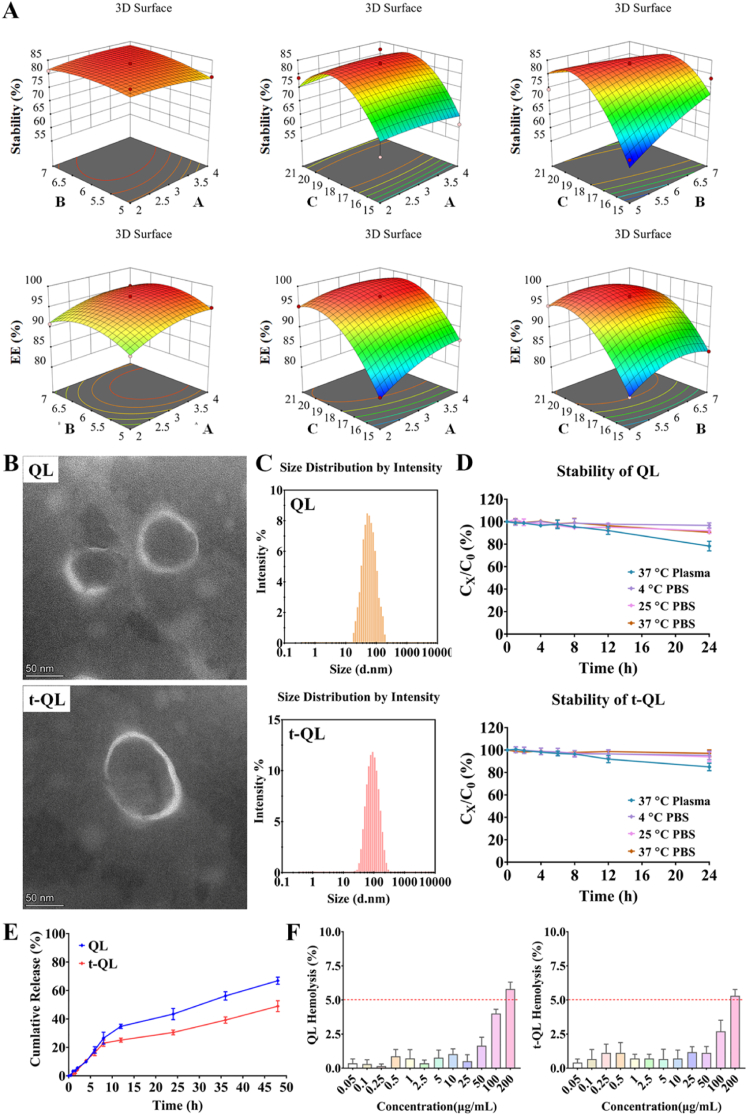
Preparation and characterization of t-QL. (A) Response surface plots (three-dimensional) showing the effect of the SPC:HSPC (X1; *w/w*), TPC:SCS (X2; *w/w*), and TPC:Q7R (X3, % *w/w*) on the Stability (%) and EE (%) in the QL; (B) Representative transmission electron microscopy (TEM) images of QL and t-QL; scale bar = 50 nm; (C) Particle sizes of QL and t-QL; (D) Stability of QL and t-QL; (E) Drug release profiles of QL and t-QL in PBS; (F) Safety evaluation about haemolysis.

As a single factor investigation, a Box–Behnken response surface method was used to optimise the formulation and preparation process of liposomes (Fig. 1A; Table S1–S3). The optimised formulation was established by analysing the response surface plots and composite desirability function, targeting the EE% and liposome stability in human plasma. The mathematical model's predictive capacity was assessed by comparison between predicted responses and five new independent experiments using the optimal conditions estimated responses on the same day. The results of stability, particle size, potential, and PDI are reported (Fig. S5A and B). The optimal dosage of DSPE-PEG2000 and incubation conditions for the cysteine-modified A20FMDV2 peptide and mal-liposomesare reported (Fig. S5C–E).

Single-factor and Box-Behnken response surface design were conducted to optimise the formulation and preparation process of Q7R liposomes. The optimal ratio of SPC : HSPC : SCS : DSPE-PEG2000 : Q7R was 136.10 : 43.90 : 28.89 : 12 : 9.83. The temperature of film forming and hydration was 55 °C, and the volume of the liposome solution system was 15 mL of PBS. In addition, the liposome solution system was subjected to ultrasonication (250 W, 240 s) under an ice bath condition. TEM also showed their morphologically regular spherical nanostructures (Fig. 1B). The particle size of QL was 66.86 ± 0.32 nm, and PDI was 0.24 ± 0.001 (Fig. 1C). The results of EE % of Q7R was 96.12 ± 3.11 % in QL, showing a good drug encapsulation efficacy. Similarly, the Zeta potential of QL was −54.07 ± 3.01 mV (Table 1). These data indicate a homogenous liposome size and absence of liposome aggregation. The stability evaluation of QL demonstrated that the liposomes have good stability in PBS and plasma.

**Table 1:** Size, PDI, Zeta potential, and content of Q7R of QL/t-QL (Mean ± SD, *n* = 3).

Sample	Particle size (nm)	PDI	Zeta potentials(mV)	EE (%)	Q7R (mg/mL)
QL	66.86 ± 0.32	0.24 ± 0.01	−54.07 ± 3.01	96.12 ± 3.11	0.634 ± 0.021
t-QL	85.32 ± 0.76	0.19 ± 0.01	−27.53 ± 1.18	95.18 ± 0.76	0.628 ± 0.005

### 3.2 Preparation and characterisation of αvβ6 integrin-targeted liposomes (t-QL)

The particle size of t-QL was 85.32 ± 0.76 nm (Fig. 1C), slightly larger than that of peptide-free liposomes, suggesting that peptide was embedded in the lipid bilayer surface. The PDI of t-QL was 0.19 ± 0.01, indicating that the dispersity of nanoparticles was relatively uniform. Similarly, TEM showed their morphologically regular spherical nanostructures. The results of EE % of Q7R in t-QL showed a good drug encapsulation efficacy; the EE % was 95.18 ± 0.76 %. In addition, the Zeta potential was −27.53 ± 1.18 mV for t-QL (Table 1), causing repulsion between the liposomes, preventing particle aggregation, and maintaining the long-term stability of the liposomes.

### 3.3 Stability assessment of t-QL

A plasma stability assessment was conducted to evaluate the *in vivo* blood compatibility of t-QL. The results showed that no significant changes in drug content were observed within 24 h, and no precipitate was observed in the testing solution system, indicating profound liposome stability in the plasma. In addition, both types of liposomes showed no visible changes in size or drug content after 24 h of incubation in PBS (pH 7.4). These findings demonstrate the potential advantages and applicability of t-QL liposomes in *in vitro/in vivo* studies (Fig. 1D).

### 3.4 In vitro release

The *in vitro* drug release behaviour was studied at 37 °C in an environment that simulated physiological conditions. The release profile showed that QL and t-QL liposomes released the drug rapidly during the first 2 h. The drug was released slightly faster during the initial 12 h, followed by a slow-release phase. The cumulative release of the drug in theinitial 12 h was approximately 40–50 %. In the subsequent period (48 h), the drug was released by a slowly sustained release, and the increase in cumulative release was only approximately 50–60 % (Fig. 1E). The peptide-modified liposomes prolonged the drug release time, i.e., the peptide protected the formulation, and the fluidity and permeability of the bilayer membrane were reduced, which delayed the release of the Q7R molecule. t-QL showed a slow drug release rate at the same period, and the total cumulative release was lower than that of QL.

### 3.5 Haemolytic activity of liposomes

To assess the potential haemolytic toxicity of t-QL, the integrity of RBCs was assessed through incubation with t-QL and QL at 0.05–200 μg/mL Q7R for 2 h. No haemolysis was observed under the tested concentrations of 0.05–100 μg/mL Q7R, and only approximately 6 % of RBCs were damaged at a high (200 μg/mL) Q7R concentration (Fig. 1F and S9). Moreover, 50 μg/mL Q7R satisfies the common clinical requirements. t-QL formulations (0.1–100 μg/mL) elicited < 5 % of total haemolysis, indicating that t-QL has good safety during intravenous injection administration and satisfies clinical requirements [32].

### 3.6 Uptake and targeting evaluation

**Fig. 2:**
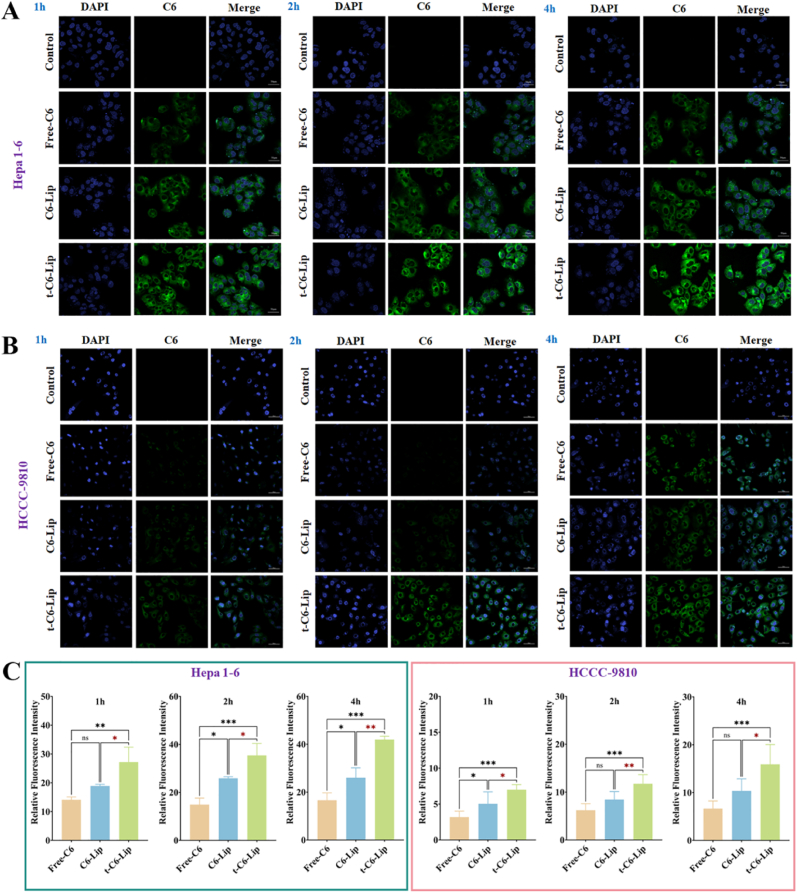
Evaluation of hepatocyte and biliary epithelial cells targeting characteristics *in vitro*. (A) LSCM fluorescence images of Heap1-6 cell line after incubation with Free-C6, C6-Lip, and t-C6-Lip for 1, 2, and 4 h; (B) LSCM fluorescence images of HCCC-9810 cell line after incubation with Free-C6, C6-Lip, and t-C6-Lip for 1, 2, and 4 h; (C) Semi-quantitative fluorescence intensity analysis of the Heap1-6 and HCCC-9810 cell line uptake of Free-C6, C6-Lip, and t-C6-Lip for 1, 2, and 4 h (*n =* 3); ∗, *p < 0.05*; ∗∗, *p < 0.01*; and ∗∗∗, *p < 0.001* compared to the Free-C6 group.

The *in vitro* uptake of t-C6-Lip in Hepa 1–6 cell (Fig. 2A) and HCCC (Fig. 2B) lines was assessed through LSCM. The amount of uptake of C6-loaded liposomes into the cytoplasm of HCCC and Hepa 1–6 cells was higher than that of free C6 after incubation for 1, 2, and 4 h at 37 °C (Fig. 2C). The results showed that the fluorescence intensities at 2 and 4 h were stronger than those at 1 h, indicating that the uptake of liposomes by HCCC and Hepa 1–6 cells increased with time, implying time dependence. Notably, most t-C6-Lip was phagocytosed by the cells and tightly dispersed in the cell membrane with some even entering the cytoplasm. However, the fluorescence intensity decreased at 4 h. Furthermore, the fluorescence intensity of t-C6-Lip was stronger than that of C6-Lip without peptide modification. This suggests that t-C6-Lip has a higher homologous targeting ability. These results suggest that peptide-modified liposomes have been successfully constructed and that the nano-delivery system has great potential for biliary epithelial cells targeting.

**Fig. 3:**
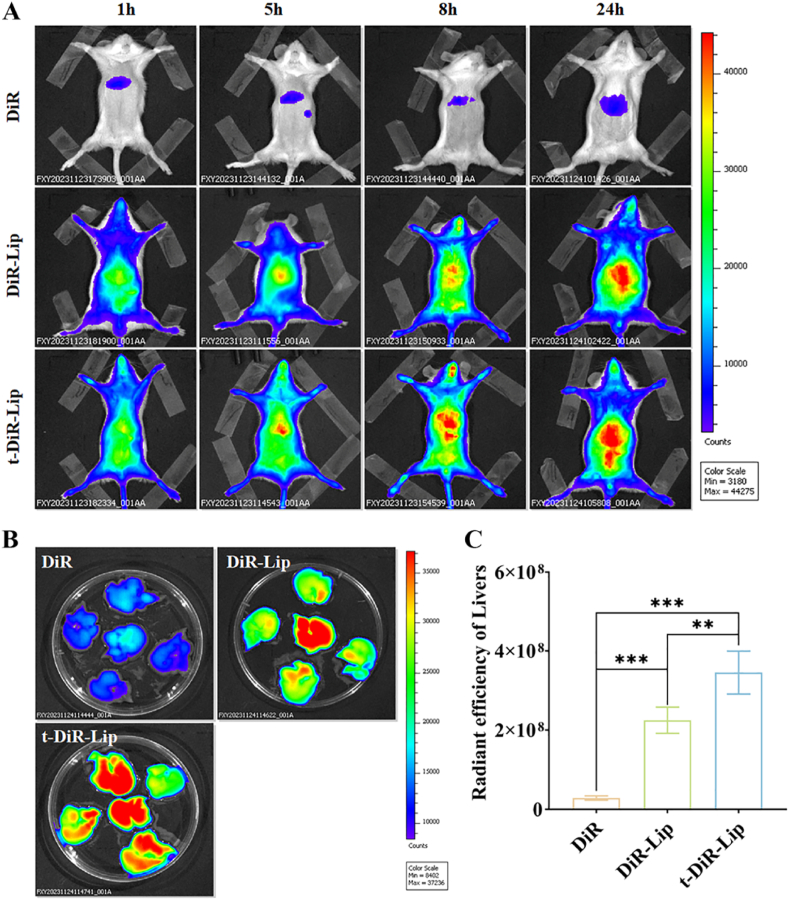
Targeting and the biodistribution of C6- t-QL in a mouse. (A) Distribution of Free-DiR, DiR-Lip, and t-DiR-Lip at 6, 12, and 24 h in mice; (B) Fluorescence images after administration of Free-DiR, DiR-Lip, and t-DiR-Lip in different tissues in mice; (C) Semi-quantitative fluorescence intensity analysis of the liver uptake of Free-DiR, DiR-Lip, and t-DiR-Lip (*n* = 3); ∗, *p < 0.05*; ∗∗, *p < 0.01*; and ∗∗∗, *p < 0.001* compared to the DiR group.

A near-infrared fluorescent tag (DiR) was used as an alternative and introduced to study the targeting ability of t-QL *in vivo* (Fig. 3A–C). The retention of DiR-Lip or t-DiR-Lip in the liver of mice was significantly higher than that of free DiR, and the liver retention in the t-DiR-Lip group was significantly higher than that in the DiR-Lip group. This may be owing to the passive hepatic targeting of liposomes and the high expression of integrin αvβ6 in bile duct endothelial cells, leading to the binding of peptide-modified liposomes to the receptor.

### 3.7 Effect of t-QL on liver protection and tocolysis

**Fig. 4:**
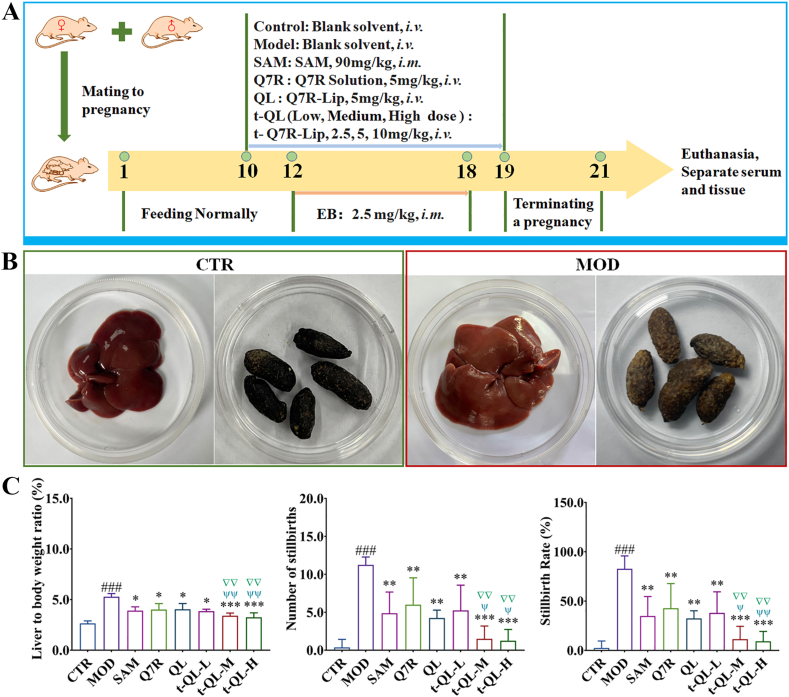
Establishment of an ICP rat model induced by EB (*n =* 8). (A) Schematic diagram of the experimental design and t-QL for treatment of ICP; (B) Representative image of livers and feces; (C) Liver index, number of stillbirths and stillbirth rate of ICP rat; ∗, *p < 0.05*; ∗∗, *p < 0.01*; and ∗∗∗, *p < 0.001* compared to the model group. ^#^, *p<0.05*; ^##^, *p<0.01;*^###^, *p<0.001* compared to the control; ▽, *p < 0.05*; ▽▽, *p < 0.01*; and ▽▽▽, *p < 0.001* compared to the Q7R; ^ψ^, *p < 0.05*; ^ψψ^, *p < 0.01*; and ^ψψψ^, *p < 0.001* compared to the QL.

**Fig. 5:**
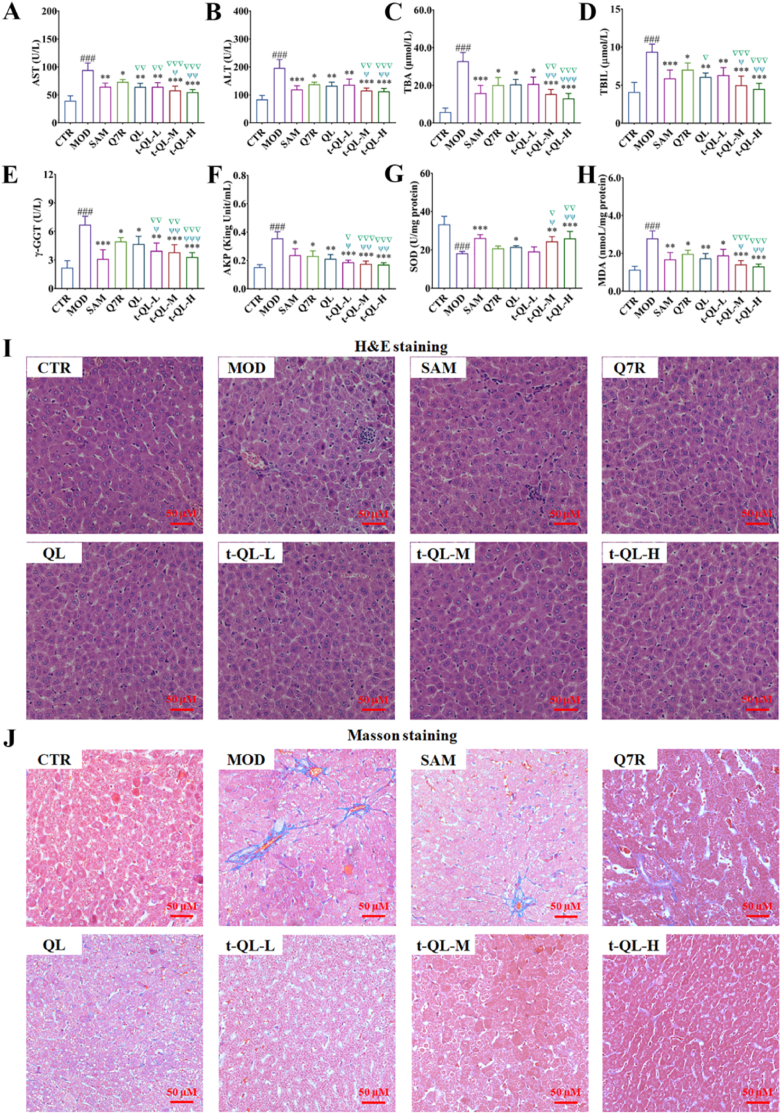
Effect of t-QL treatment on ICP. (A–H) Serum ALT, AST, and ALP levels of ICP rat after treatment with different concentrations of t-QL (*n =* 8); ∗, *p < 0.05*; ∗∗, *p < 0.01*; and ∗∗∗, *p < 0.001* compared to the model group. ^#^, *p<0.05*; ^##^, *p<0.01;*^###^, *p<0.001* compared to the control; ▽, *p < 0.05*; ▽▽, *p < 0.01*; and ▽▽▽, *p < 0.001* compared to the Q7R; ^ψ^, *p < 0.05*; ^ψψ^, *p < 0.01*; and ^ψψψ^, *p < 0.001* compared to the QL; (I) Representative images of H&E stained liver tissue of ICP rat after treatment with different concentrations of t-QL, scalebar = 50 μm; (J) Representative images of Masson-stained liver tissue of ICP rat after treatment with different concentrations of t-QL, scalebar = 50 μm.

The feces and liver exhibited noticeable jaundice, indicating that the ICP rat model was successfully established (Fig. 4A and B). The liver index of the ICP model group was higher than that of the control group. The liver indices of the positive control, free-Q7R, QL, and t-QL groups were significantly decreased (*p<0.05*) compared with those of the model group (Fig. 4C). In addition, the stillbirth rate (Fig. 4C) was significantly higher in the model group and decreased after administering t-QL (*p<0.05*). The intervention effects of the medium- and high-dose t-QL groups were the most significant. Biochemical analysis, conducted to evaluate the therapeutic effects of t-QL, showed that serum ALT, AST, AKP, TBA, TBIL, and γ-GGT levels in the model group were significantly increased (*p<0.05*) (Fig. 5A–H). In contrast, after t-QL treatment, this status was significantly reversed, particularly in the medium- and high-dose groups. These results indicate that Q7R protects the liver and exerts tocolytic effects. More significantly, through the optimisation of the drug delivery system and targeted modification, t-QL exhibited an excellent therapeutic effect.

### 3.8 t-QL alleviated pathological changes in the liver of ICP-modelled rats

H&E staining showed that the hepatocyte structure in the control group was normal, and the cell nuclei were clearly visible without cell necrosis or inflammatory cell infiltration (Fig. 5I). In contrast, hepatocytes in the ICP model group exhibited variable sizes and loose cytoplasm. Similarly, the liver cells showed focal necrosis, eosinophilic changes, and punctate necrosis, along with infiltration of inflammatory factors in the liver tissue. The cells in the positive control, QL, and t-QL groups were significantly lighter than those in the model group, and the free-Q7R group also improved. These findings show that t-QL effectively improved liver tissue cell damage. Masson staining showed that the liver sections in the control group had intact cell structures, with no noticeable blue collagen fibre deposition (Fig. 5J). However, the liver tissue sections of the ICP model group displayed abundant blue collagen fibre deposition in the liver indicating fibrosis induced by cholestasis.

QL and t-QL treatments significantly attenuated the degree of hepatic putrescence, fibrosis, and inflammatory cell infiltration. These results indicate that QL and t-QL protected against EB-induced liver cholestasis-related fibrosis in ICP rats.

### 3.9 Morphological and functional evaluation of the liver mitochondria

**Fig. 6:**
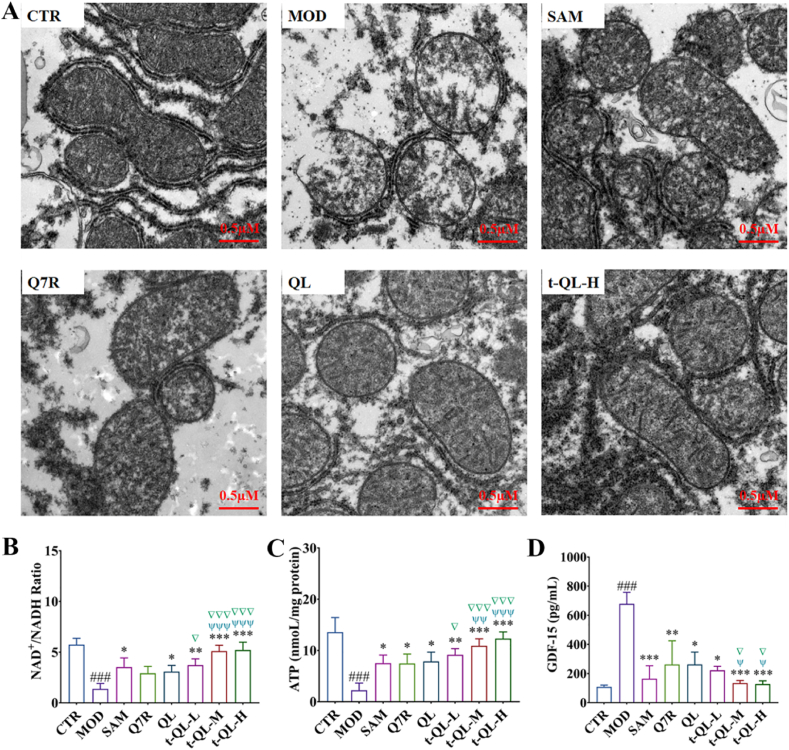
Effect of t-QL on mitochondrial structure and function of ICP rat. (A) TEM images of hepatocyte mitochondrial, scalebar = 0.5 μm; (B) Liver NAD^+^/NADH ratio; (C) ATP levels and (D) GDF-15 levels in CTR and ICP rat were measured after treatment (*n =* 8). ∗, *p < 0.05*; ∗∗, *p < 0.01*; and ∗∗∗, *p < 0.001* compared to the model group; ^#^*p<0.05;*^###^, *p<0.001* compared to the control; ▽, *p < 0.05*; ▽▽▽, *p < 0.001* compared to the Q7R; ^ψ^, *p < 0.05*; ^ψψ^, *p < 0.01*; ^ψψψ^, *p < 0.001* compared to the QL.

TEM showed that, in the control group, liver cell mitochondria were structurally intact with a clear cristae structure and abundant mitochondrial matrix (Fig. 6A). However, the mitochondria in the model group were swollen and deformed or ruptured, and the inner cristae almost disappeared. The morphology of the mitochondria of the liver cells of the model animals was significantly improved by t-QL administration, with the swelling reduced and the inner cristae arranged clearly, indicating that t-QL exerts its therapeutic effect in ICP by improving the mitochondrial structure.

The biochemical indicators of the mitochondrial function were also evaluated using commercial reagent kits to analyse the therapeutic effects of t-QL (Fig. 6B–D). The results showed that the MDA and GDF-15 levels in the model group were significantly increased (*p<0.001*), and the levels of SOD, ATP, and NAD^+^/NADH ratio were significantly decreased (*p<0.001*). In contrast, the levels of these parameters were improved significantly (*p<0.05*) in the positive control and the different Q7R-treatment groups. These results confirm that t-QL maintains normal mitochondrial morphology and improves oxidative stress and energy metabolismrelated to mitochondrial function.

### 3.10 t-QL ameliorated changes in enzymes involved in mitochondrial function and BA synthesis, metabolism, excretion

**Fig. 7:**
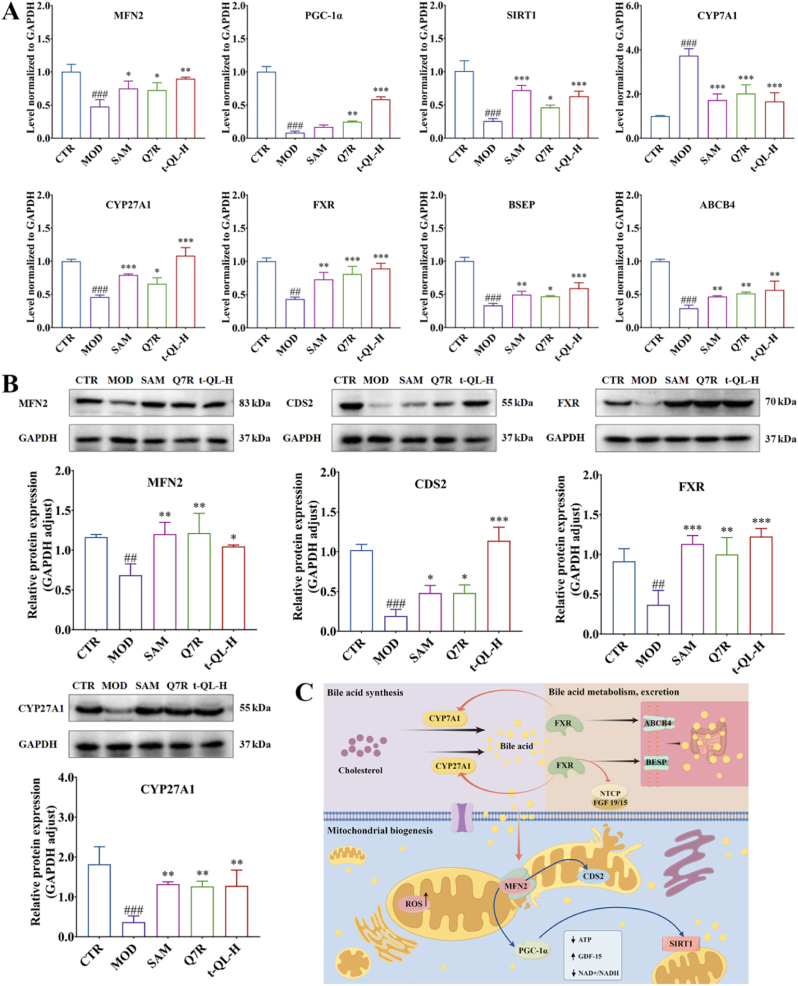
Effects of t-QL on mRNA and protein expression in the liver of ICP rat. (A) Analysis of mRNA level are related to bile acid metabolism and mitochondria metabolism in the liver; (B) Analysis of proteins are related to bile acid metabolism and mitochondria metabolism in the liver, (*n* = 3), ∗, *p < 0.05*; ∗∗, *p < 0.01*; and ∗∗∗, *p < 0.001* compared to the model group; ^#^*p<0.05;*^###^, *p<0.001* compared to the controlgroup; (C) The brief diagram of bile acid metabolism and mitochondria metabolism pathway.

The mRNA level of CYP7A1 was increased in the liver of vehicle-treated ICP model rat compared with that in normal rats (Fig. 7A). Conversely, the expression of Sirtuin 1 (SIRT1), MFN2, peroxisome proliferator-activated receptor γ coactivator 1α (PGC-1α), FXR, CYP27A1, bile salt export pump (BSEP), and ABCB4 showed a decreasing trend in the vehicle-treated ICP model rat. t-QL significantly modulated the expression of genes towards that in normal animals. In addition, the FXR, CYP27A1, MFN2 and CDS2 protein levels decreased in these ICP animals (Fig. 7B). These findings indicate that mitochondrial function, BA synthesis, metabolism and excretion, and even downstream enzymes involved in mitochondrial dysfunction, oxidative stress, inflammation, and cholestasiswere altered in ICP model rats (Fig. 7C), but that these alterations were attenuated by t-QL treatment.

### 3.11 t-QL regulated BA metabolism

**Fig. 8:**
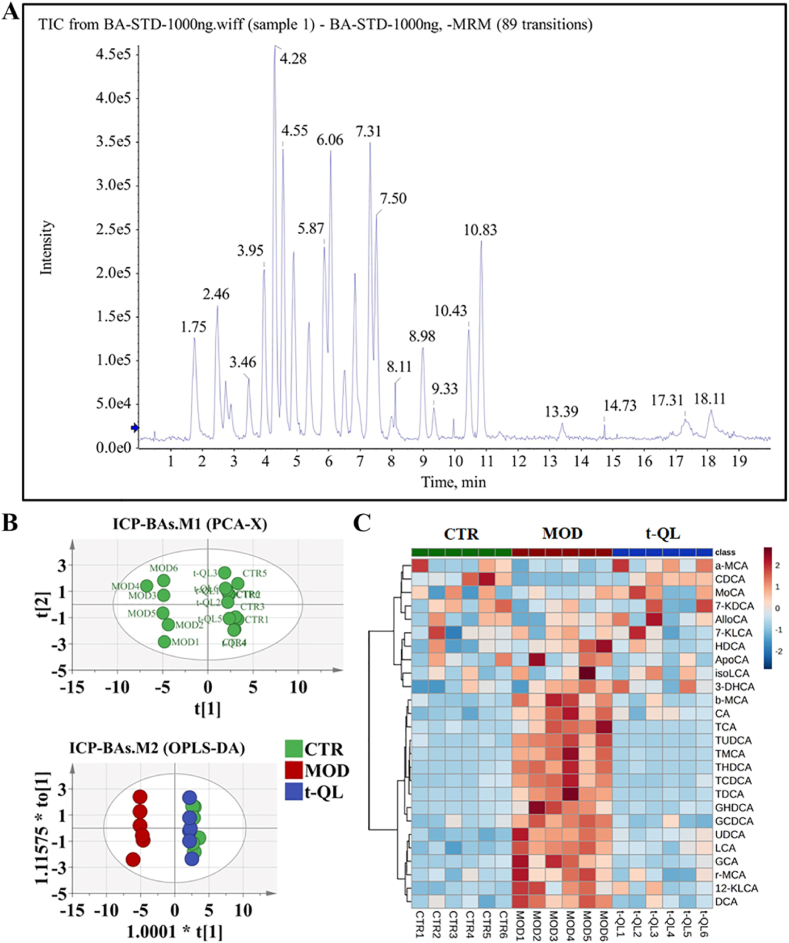
Effect of t-QL on bile acid metabolism in ICP rat (*n* = 6). (A) Representative UPLC-MS/MS chromatogram of bile acid detection; (B) PCA-X and OPLS-DA score plot of ICP model rat; (C) Heatmap analysis of ICP model rat.

BA synthesis and decomposition are crucial in disease processes. The BA levels reflect the degree of liver injury caused by hepatotoxicity and cholestasis. In this study, a highly sensitive UPLC-MS/MS method was conducted to assess 26 BAs in the serum (Fig. 8A, S10). The results showed that 16 BA levels were significantly increased in ICP-modelled rats, including TCDCA, taurocholicacid (TCA), taurodeoxycholic acid (TDCA), THDCA, TUDCA, TMCA, glycochenodeoxycholate (GCDCA), glycocholic acid (GCA), GHDCA, LCA, UDCA, DCA, CA, α-MCA, β-MCA, and 12-KLCA, with decreased CDCA levels. After the Q7R intervention, the BA levels converged to normal. The PCA, OPLS-DA score plot showed that the control, drug intervention, and model groups could be significantly differentiated (Fig. 8B–S11A–C), PCA–X analysis showed R2X(cum) = 0.635 and Q2(cum) = 0.407; OPLS–DA analysis showed R2X(cum) = 0.627 and R2Y(cum) = 0.497, Q2(cum) = 0.387. The results showed that the PCA model had good predictive ability. The heatmap results clearly responded to the trend of the samples' BA profiles (Fig. 8C). Combined with the VIP analysis and one-way ANOVA, 14 BAs, including CA, DCA, GCDCA, GCA, GHDCA, LCA, β-MCA, TMCA, TCDCA, TCA, TDCA, THDCA, TUDCA, and UDCA were screened as potential markers of ICP, which can be used to assess the ICP extent and treatment efficacy (Figure S11 D).

**Fig. 9:**
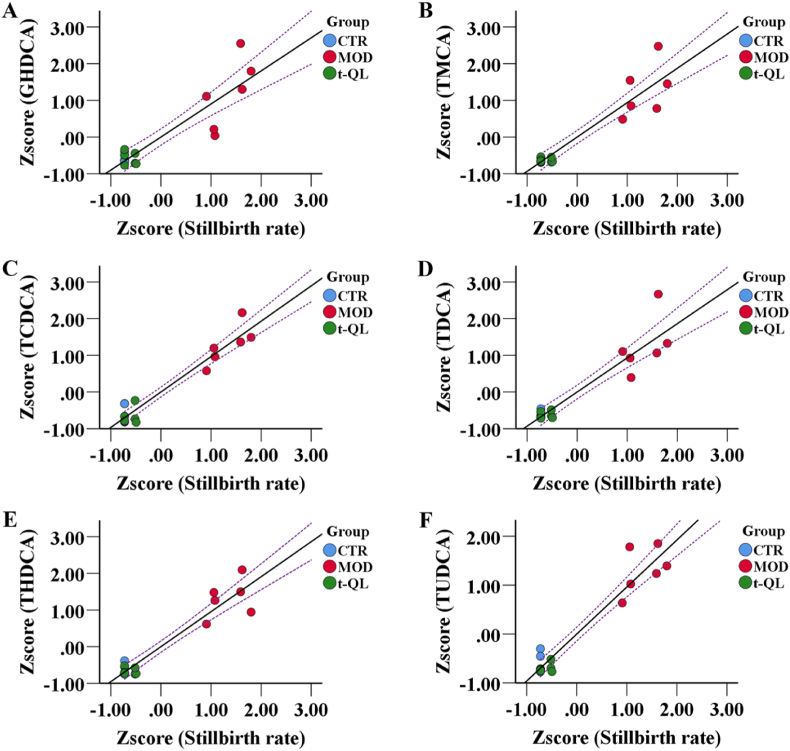
Bile acid metabolite correlations with stillbirth rate scores (*n* = 6). Scatter plot correlations between (A) serum GHDCA and stillbirth rate scores; (B) serumTMCA and stillbirth rate scores; (C) serum TCDCA and stillbirth rate scores; (D) serum TDCA and stillbirth rate scores; (E) serum THDCA and stillbirth rate scores; (F) serum TUDCA and stillbirth rate scores; dashed lines indicate the mean value confidence interval for the line of best fit.

The stillbirth rate is the primary outcome index in ICP. To explore the correlation between the stillbirth rate and serum BA level, we estimated the association between the adjusted Z scores of the serum levels of the 15 BAs and the stillbirth rate (cutoff value of Pearson correlation > 0.9). In healthy pregnant rats, ICP rats, and t-QL treatment rats, the serum levels of GHDCA, TMCA, TCDCA, TDCA, THDCA, and TUDCA were significantly associated with higher stillbirth rate scores (R = 0.902, *P = 0.000*, Fig. 9A; and R = 0.937, *P = 0.000*, Fig. 9B; and R = 0.965, *P = 0.000*, Fig. 9C; and R = 0.931, *P = 0.000*, Fig. 9D; and R = 0.954, *P = 0.000*, Fig. 9E; and R = 0.962, *P = 0.000*, Fig. 9F, respectively). This result suggests a potential correlation between the levels of these six bile acids and the stillbirth rate; however, their more direct causal relationship with stillbirth warrants further investigation.

In brief, we systematically evaluated the modulating effect of Q7R and t-QL on the BA profile of the ICP rat model, which is useful for the screening of characteristic BA species for the diagnosis and differentiation of ICP, as well as for the evaluation of the efficacy of drugs in ICP treatment.

## 4 Discussion

ICP is the most prevalent pregnancy-associated liver disease; however, its etiopathogenesis remains indeterminate. The estimated prevalence of ICP ranges from 0.2 % to 25.0 % [33,34], and the potential pathogenic factors of ICP include hormonal secretion levels, patient genetics, immune function, and environmental conditions [35].

UDCA and SAM are common ICP treatment agents; however, even the largest trials have shown minimal benefits regarding stillbirth, preterm birth, and neonatal unit admission outcomes [36,37]. Thus, identifying novel therapeutic drugs and drug delivery systems for effective delivery to diseased liver sites is urgent. In this study, the formulation we designed was administered intravenously. To maintain the same stimulation on experimental animals, SAM was selected as the positive control drug because it is suitable for intramuscular/intravenous administration.

Natural compounds, including Q7R, paeoniflorin, and geniposide, have recently been shown to have hepatoprotective and choleretic effects in ICP and cholestasis, and use of nanoparticle delivery systems for these small-molecule chemical compounds targeting the liver have several innate advantages [[38], [39], [40]]. We used a combination of the lipids HSPC, SPC, sodium cholesterol sulphate, and DSPE- PEG2000-Mal for the formation of liposomal vesicles via the thin film dispersed method, and the peptide A20FMDV2 with thiol residue was used to conduct derivative reaction with DSPE-PEG2000-Mal on the surface of liposomes to facilitate liver targeting. *In vitro* cytotoxicity and haemolysis tests supported the safe use of liposomal formulations for disease treatment. Cell uptake and small-animal *in vivo* imaging analyses confirmed that the nanoparticle could stabilise its cargoes *in vivo* and release Q7R accurately in the liver region, improving the targeting and efficacy of the drug. Notably, the saturated solubility of Q7R in pure water was approximately 0.0041 ± 0.0001 mg/mL. Notably, when formulated into liposomes, the Q7R content reached 0.5633 ± 0.0115 mg/mL (Fig. S12), the biological activity of Q7R was improved significantly by using liposomes as carriers.

The oestradiol 17β-D-glucuronide (E17G) produced from oestradiol in the liver owing to a conjugation reaction is considered primarily responsible for ICP. Salas confirmed the participation of oxidative stress in ICP and ROS through internalisation of canalicular transporters, such asmultidrug resistance-associated transporter 2, partially mediated E17G-induced cholestasis [41,42]. Oxidative stress levels were significantly increased inthe bile duct ligation-induced chronic liver injuryrat model, and liver mitochondrial indices of functionality deteriorated in the animal model. The mitochondrial ATP content was significantly decreased, whereas mitochondrial ROS formation and swelling were increased. The accumulation of BAs affects the mitochondria, causing oxidative stress, mitochondrial permeability transition, dissipating mitochondrial membrane potential, inhibiting mitochondrial respiratory complexes, and finally the release of cell death mediators from the mitochondria [43,44].

Current evidence indicates that BA play key regulatory roles in mitochondrial activity, mitochondrial gene dysregulation occurs in ICP, concomitant with elevated systemic oxidative stress. Specifically, TCDCA demonstrates cytotoxicity in mouse primary hepatocytes by inducing mitochondrial permeability transition. In ICP cell models, both mitochondrial morphology and function are significantly disrupted, elevated serum BA levels in ICP patients may propagate mitochondrial dysfunction in placental trophoblasts via NRF1/PGC-1α mechanism. Collectively, these findings establish an intricate pathophysiological interconnection between BA homeostasis and mitochondrial integrity [[45], [46], [47]].

The metabolic function of liver mitochondria was impaired during cholestasis. Rats with long-term cholestasis and patients with ICP exhibited disordered benzoic acid metabolism, which reflects the hepatic mitochondrial function [48,49]. PGC-1α serves as a pivotal transcriptional coactivator involved in the regulation of mitochondrial dynamics and is recognised as a significant downstream target of SIRT1. PGC-1α and SIRT1 are implicated in ameliorating oxidative stress, as well as enhancing mitochondrial architecture and function [50]. Therefore, in this study, we focused on investigating the effects of Q7R on the liver mitochondrial structure and function in the ICP animal model. We found that the Q7R liver-targeted liposomes significantly improved the morphology and ridge structure of the mitochondria, as well as the ATP, GDF-15, NAD^+^/NADH ratio, and expression levels of PGC-1α, SIRT1, MFN2 related to mitochondrial function. To our knowledge, this is the first time theseobservations on Q7R have been reported. From these results, we speculate that improved mitochondrial damage repair progression might be the primary potential mechanism of Q7R in treating ICP.

In humans, CYP7A1, CYP27A1, andsterol 12a-hydroxylase (CYP8B1) regulate the synthesis of the primary BAs, including CDCA and CA. BA biosynthesis terminates the conjugation of glycine or taurine, generating taurine- or glycine-conjugated BAs, such as glycochenodeoxycholate (GCDCA), taurochenodeoxycholate (TCDCA), glycocholic acid (GCA), taurocholic acid (TCA), glycolithocholic acid (GLCA), and taurodeoxycholic acid (TDCA) in humans. Additional forms of BA conjugation include sulfation, hydroxylation, and glucuronidation [51,52]. BSEP and ABCB4 play crucial roles in facilitating the secretion of BAs into the bile canaliculi, thereby ameliorating cholestasis and reducing cytotoxicity.

In the pathophysiological process of ICP, the synthesis of BAs is increased, while their transport is impaired. Higher oestrogen and progesterone levels in the late stages of pregnancy inhibit BA flow through the liver. Similarly, 17β-oestradiol, E17G, and allopregnanolone sulphate inhibit the bile salt export pump, affecting the biliary secretion of BAs, which are induced by cholestasis [53]. An untargeted metabolomics study showed that plasma GCDCA, TCDC, GCA, TC, GLCA, and TDCA levels were increased in the ICP group [54]. Xiong et al. developed a chemical derivatisation-based LC-MS/MS method and successfully determined 107 BAs in the serum of patients with ICP. The analysis revealed the BA profile with decreased unconjugated, sulphate, and doubly conjugated BAs and increased glycine and taurine conjugates [55].

In this study, we investigated the effect of Q7R on the BA metabolism profile in ICP rat models. GHDCA, TMCA, TCDCA, TDCA, THDCA, and TUDCA were selected as characteristic biomarkers for ICP, which can be used to evaluate ICP severity and the efficacy of hepatoprotective and choleretic drugs such as Q7R in treating ICP.

Previous studies have revealed that the gut microbiome differs significantly between individuals with ICP and healthy pregnant women. *B. fragilis*-mediated FXR signalling inhibition was responsible for excessive BA synthesis and interrupted hepatic bile excretion to promote ICP initiation [56]. *Roseburia intestinalis*is the key bacterial species for improved phenotypes associated with ICP through the BA/FXR-fibroblast growth factor 15 signalling pathway [31]. However, this field was not included in thisstudy, indicating the need for further investigation.

Our study has some limitations. First, we verified the key role of the structure and function of the mitochondria in ICP treatment; however, since MFN2 levels are related to the function of mitochondrial signalling, further supplementing agonists or antagonists of MFN2 on the ICP model for verification would make the experimental results more rigorous and convincing. Second, recognised ICP cell models remain lacking, and further exploration is essential. Finally, *in vivo* peptide A20FMDV2 stability and the protein corona effect may decrease *in vivo* targeting efficiency, which also needs to be investigated further.

## 5 Conclusions

In this study, we investigated the formulation of a targeted liposomal intravenous drug delivery system to directly deliver Q7R to the liver for treating ICP. Q7R was trapped in the phospholipid bilayer of the liposomes for efficient delivery at the target site, and active targeting was confirmed *in vitro* in Hepa 1–6 and HCCC cell lines.

The efficacy of this liposomal formulation was validated in an ICP rat model to assess its effects on stillbirth rates and BA levels, with previous studies indicating that Q7R has beneficial effects on cholestasis. For the first time, this study validated the use of injection liposomal formulation as a novel drug delivery method to the liver for improving ICP with Q7R. We conclude that the Q7R delivered via the liposomes modified with targeted peptides improved mitochondrial structure and function, regulating BA metabolism in the ICP rat model, and enriched several molecular signalling pathways involved in ICP pathophysiology.

## CRediT authorship contribution statement

**Xiaoying Feng:** Writing – review & editing, Writing – original draft, Project administration, Methodology, Data curation. **Ling Dai:** Writing – review & editing, Writing – original draft, Visualization, Validation, Project administration. **Yanfang Guo:** Data curation, Methodology, Validation. **Liuting Zhong:** Supervision, Project administration, Investigation. **Yuxiu Zheng:** Writing – review & editing, Validation, Formal analysis. **Senling Feng:** Writing – review & editing, Supervision, Project administration. **Liping Cao:** Writing – review & editing, Supervision, Project administration, Investigation, Funding acquisition, Conceptualization. **Zhongwen Yuan:** Writing – review & editing, Visualization, Validation, Supervision, Software, Resources, Project administration, Methodology, Investigation, Funding acquisition, Formal analysis, Data curation, Conceptualization.

## Data statement

All data from this study is available from the corresponding authors upon reasonable request.

## Funding

This work was supported by the 10.13039/501100001809National Natural Science Foundation of China [grant number 81803689]; the Major Scientific and Technological Projects in Traditional Chinese Medicine of Guangzhou [grant number 2025CX014]; the Guangzhou Municipal Science and Technology Project [grant number 2024A03J0187]; the YIWEN Talent Project of The Third Affiliated Hospital of Guangzhou Medical University [grant number 2021#9]; the Research Fund for Lin He Academician New Medicine [Grant Number. 2021HLKY10], and plan on enhancing scientific research in GMU.

## Declaration of competing interest

The authors declare that they have no known competing financial interests or personal relationships that could have appeared to influence the work reported in this paper.

## Data Availability

Data will be made available on request.

## Glossary

AKP: alkaline phosphatase
ALT: alanine transaminase
AST: aspartate transaminase
BA: bile acid
CDCA: chenodeoxycholic acid
E17G: oestradiol 17β-D-glucuronide;
EB: oestradiol benzoate
EE %: entrapment efficiency
FXR: farnesoid X receptor
GCA: glycocholic acid
GCDCA: glycochenodeoxycholate
GDF-15: growth differentiation factor 15
GLCA: glycolithocholic acid
H&E: haematoxylin and eosin
HPLC: high-performance liquid chromatography
ICP: intrahepatic cholestasis of pregnancy
LSCM: laser confocal scanning microscopy
MDA: malondialdehyde
PBS: phosphate-buffered solution
PCA: principal component analysis
PDI: polydispersity index
Q7R: quercetin 7-rhamnoside;
SOD: superoxide dismutase
TBA: total bile acid
TBIL: total bilirubin
TCA: taurocholic acid
TCDCA: taurochenodeoxycholate
TDCA: taurodeoxycholic acid
TEM: transmission electron microscopy
UDCA: ursodeoxycholic acid
γ-GGT: gamma-glutamyl transferase
