# Supplementary material for: An integrin-based quercetin 7-rhamnoside liver-targeted delivery liposomes for intrahepatic cholestasis in pregnancy
Source: Mater Today Bio. 2025 Jun 27;33:102031. doi: 10.1016/j.mtbio.2025.102031 (PMC12268842; doi:10.1016/j.mtbio.2025.102031)
Supplement: Multimedia component 1 [file mmc1.docx]

**Supplementary Material**

**An integrin-based quercetin 7-rhamnoside liver-targeted delivery liposomes for intrahepatic cholestasis in pregnancy**

**1. Characterization of cysteine-modified A20FMDV2 peptide**


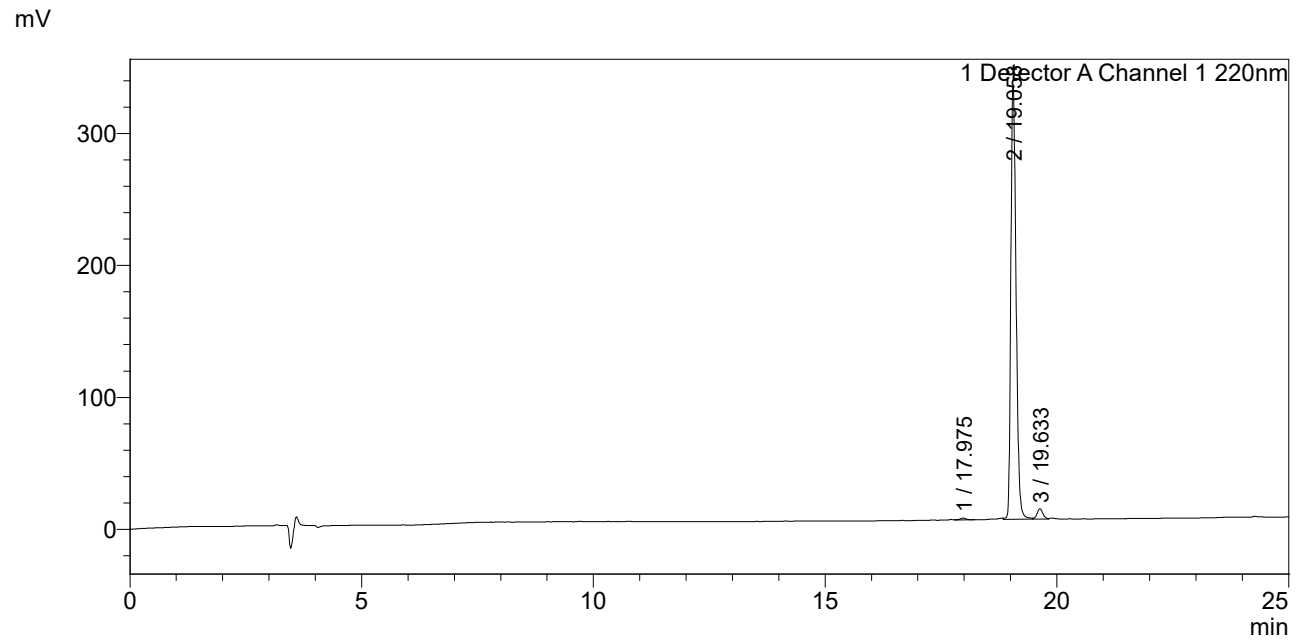


Figure S1. HPLC chromatogram of cysteine-modified A20FMDV2 peptide.


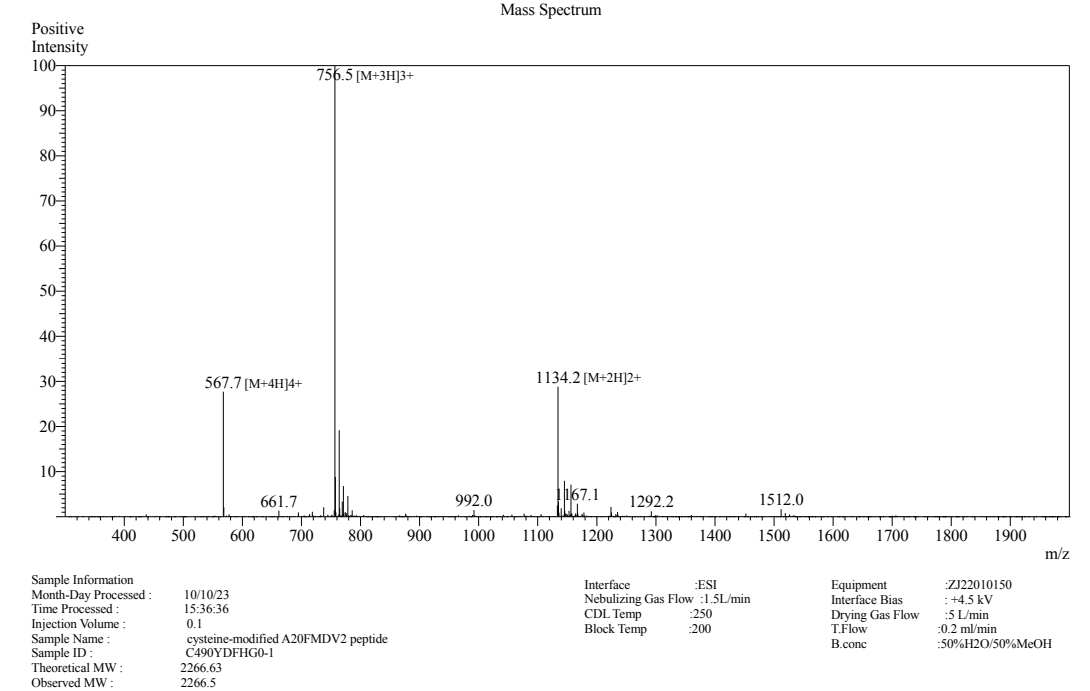


Figure S2. MS spectrum of cysteine-modified A20FMDV2 peptide.

**2. Liposome prescription and preparation process optimization**

Single factor investigation involve in SPC/HSPC ratio, TPC/SCS ratio, Q7R/ TPC ratio, ultrasonic power, ultrasonic time were carried out to evaluate the key factor on preparation of Q7R liposome. The results of stability, particle size, potential and PDI were shown in Figure S1 A, B and Figure S2. Single factor investigation indicated that SPC/HSPC ratio, TPC/SCS ratio, Q7R/ TPC ratio are the main influencing factor.

On this basis of single factor investigation, an [Box-Behnken](https://www.so.com/link?m=zhSE3elo50CFdgSXNkxIYWdfYM71jiyzAQ%2FzyJ2K3oRrB%2FmIpByKjZZ58sOgix6DG%2FcwwKoB%2B11tVYokvqI0dPTvMqe2s9crF5ZLQp3WEcOMaOEkhfT52m6DGma%2Fb2R9NK%2F4NDSQlqdeqXE6pAszPNvcouspuutqr5JqKPIoRzh6MM%2FkcQ%2FFOz6k1Sps%3D) response surface method was designed, in order to optimized the formulation of liposome, the [Box-Behnken](https://www.so.com/link?m=zhSE3elo50CFdgSXNkxIYWdfYM71jiyzAQ%2FzyJ2K3oRrB%2FmIpByKjZZ58sOgix6DG%2FcwwKoB%2B11tVYokvqI0dPTvMqe2s9crF5ZLQp3WEcOMaOEkhfT52m6DGma%2Fb2R9NK%2F4NDSQlqdeqXE6pAszPNvcouspuutqr5JqKPIoRzh6MM%2FkcQ%2FFOz6k1Sps%3D) response surface design table as show in Table S3, S4. The optimized formulation was established by analysing the response surface plots and the composite desirability function, targeting the EE% and the stability of liposome in human plasma, The mathematical model’s predictive capacity was assessed by comparison between predicted responses and new five independent experiments using the optimal conditions estimated responses on the same day. The results of stability, particle size, potential and PDI were shown in Figure S5A, B. The prescription ratio after optimization is HSPC : SPC : SCS : Q7R= 43.90 : 136.10 : 28.89 : 9.83. Prepare Q7R liposome using the optimal ratio, the predicted values obtained from the response optimization of Box–Behnken design are consistent with the experimental values (Table S3).

Subsequently, the optimal dosage of DSPE-PEG2000 was determined based on the stability, particle size, zeta potential, and PDI of the liposome. The experimental results indicated that 12 mg of DSPE-PEG2000 was appropriate for the preparation of liposome (Figure S5 C).


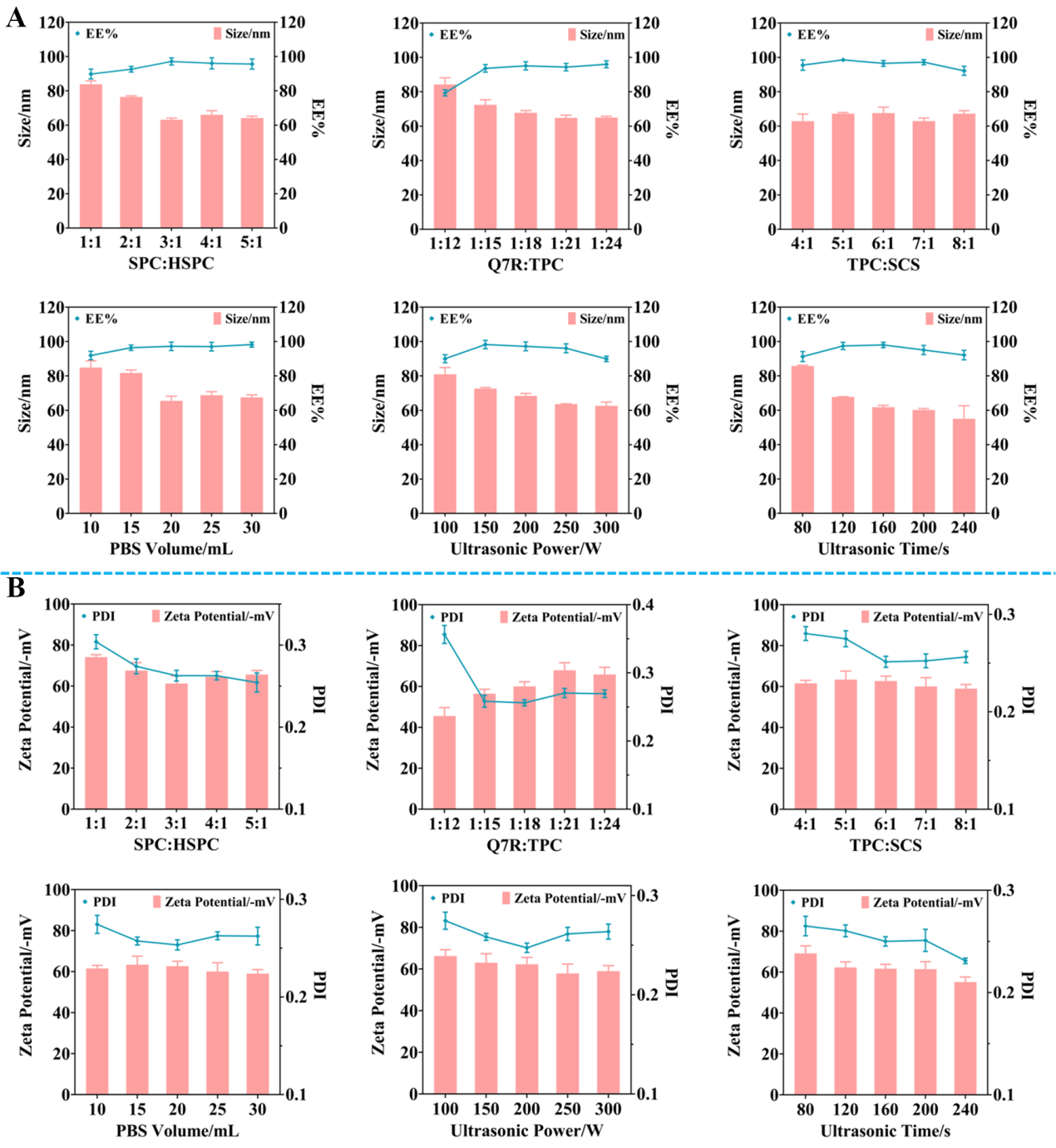


Figure S3. Characterization evaluation of QL in single factor experiment.

(A) Particle sizes and EE%; (B) PDI and Zeta potential.

Note: TPC represents the total sum of the quality of HSPC and SPC.

**
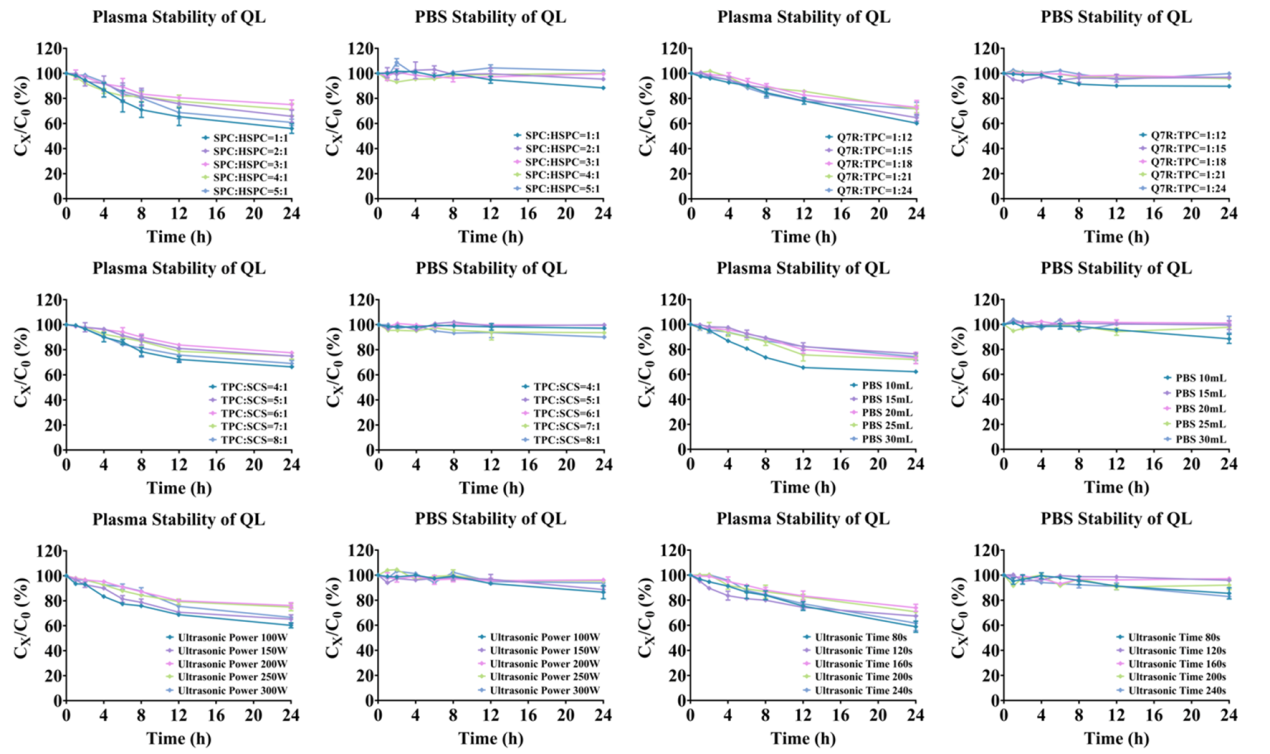
**

Figure S4. Stability of QL in PBS or 50% plasma at 37 °C in single factor experiment.

Table S1. Investigated factors and factor levels of Box-Behnken experimental design.

| Factor | Level | | |
| --- | --- | --- | --- |
|  | Low Level (-1) | Intermediate Level (0) | High Levle (+1) |
|  |  |  |  |
| A (SPC:HSPC, *w/w*) | 2:1 | 3:1 | 4:1 |
| B (TPC:SCS, *w/w*) | 5:1 | 6:1 | 7: 1 |
| C (TPC:Q7R, *w/w*) | 15:1 | 18:1 | 21:1 |

Table S2. Box-Behnken Design arrangement and experimental values for each response for QL.

| Run | Factor A | Factor B | Factor C | Stability (%) | EE (%) |
| --- | --- | --- | --- | --- | --- |
| 1 | -1 | -1 | 0 | 83.4847 | 90.2036 |
| 2 | 1 | 0 | 1 | 81.8812 | 96.0519 |
| 3 | 1 | 1 | 0 | 80.9519 | 95.1204 |
| 4 | -1 | 0 | 1 | 78.6003 | 95.1683 |
| 5 | 0 | -1 | -1 | 60.1805 | 80.8472 |
| 6 | 0 | -1 | 1 | 74.4174 | 95.2827 |
| 7 | 0 | 0 | 0 | 83.8204 | 96.9241 |
| 8 | 0 | 1 | 1 | 68.1660 | 93.7259 |
| 9 | 1 | 0 | -1 | 61.3128 | 86.8821 |
| 10 | 0 | 0 | 0 | 83.7615 | 97.1183 |
| 11 | 1 | -1 | 0 | 78.9700 | 94.8542 |
| 12 | 0 | 1 | -1 | 78.4291 | 84.0044 |
| 13 | -1 | 0 | -1 | 61.1804 | 80.8175 |
| 14 | 0 | 0 | 0 | 82.5129 | 97.5908 |
| 15 | -1 | 1 | 0 | 81.1537 | 90.8700 |

Table S3. Predicted and experimental values obtained from the response optimization of Box–Behnken design.

| Responses | Predicted value | Experimental value |
| --- | --- | --- |
| Stability (%) | 83.36 | 81.68±3.23 |
| EE (%) | 97.21 | 98.03±1.18 |

**
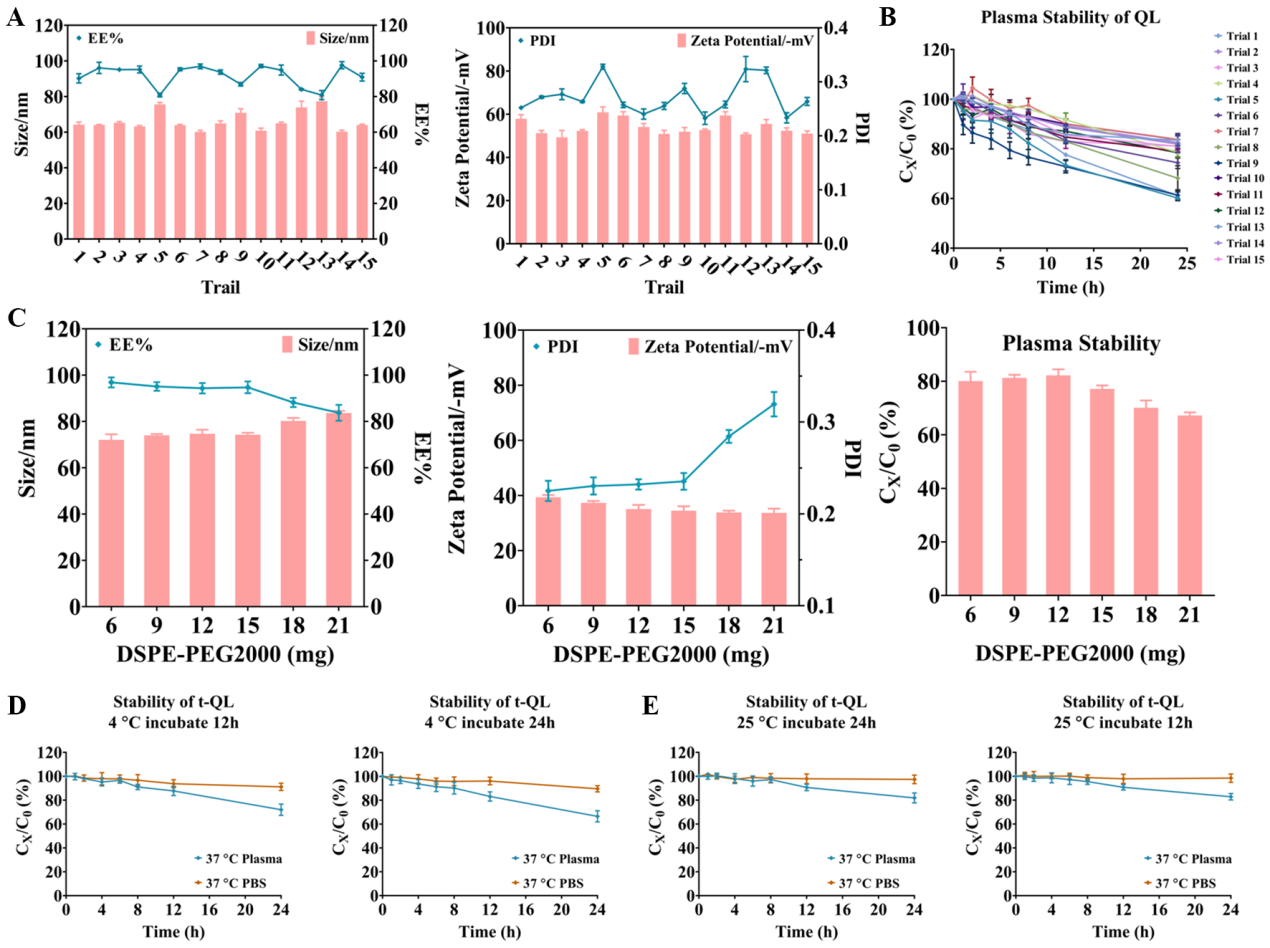
**

Figure S5. Box–Behnken design experiment of QL and optimization of t-QL incubation conditions.

(A) Particle sizes, PDI, EE% and Zeta potential of QL in Box–Behnken design experiment;

(B) Stability of QL in 50% plasma at 37 °C;

(C) Investigation of the optimal dosage of DSPE-PEG2000;

(D) Stability of t-QL performed at 4 °C incubation conditions with peptide A20FMDV2 in PBS and 50% plasma at 37 °C;

(E) Stability of t-QL performed at 25 °C incubation conditions with peptide A20FMDV2 in PBS and 50% plasma at 37 °C.

**3. Optimisation of peptide loading on liposome surface**

The incubation conditions for the cysteine-modified A20FMDV2 peptide and Mal-liposome were evaluated by assessing the stability of the liposome in human plasma. The preferred incubation conditions are presented in Figure S5 D, E. According to the preparation method of Q7R liposome, 200 μL of different peptide concentrations (5, 10, and 20 mg/mL for the low, medium, and high dosages, respectively) were added to 4.8 mL of C6 liposome, and the liposome were shaken at 25 °C for 24 h (Preferred incubation conditions, The results are shown in Figure S 5D, E). Dialysis was conducted to remove free peptides overnight at 4 °C. The three groups of t-C6-liposomes (t-C6-Lip) finally obtained were used in the fluorescence uptake experiments in HCCC-9810 cells.

The final peptide A20FMDV2 load on the liposome surface was investigated through a cell uptake experiment of C6. The results showed that HCCC-9810 cells exhibited better uptake efficiency, accompanied by an increase in peptide A20FMDV2 concentration from 0.2 to 0.4 mg/mL of liposome solution. There were no significant differences between 0.4 and 0.8 mg peptide A20FMDV2 per mL of the liposome solution. Therefore, we used 0.4 mg peptide per mL of liposome to prepare the t-QL for subsequent studies (Figure S6).


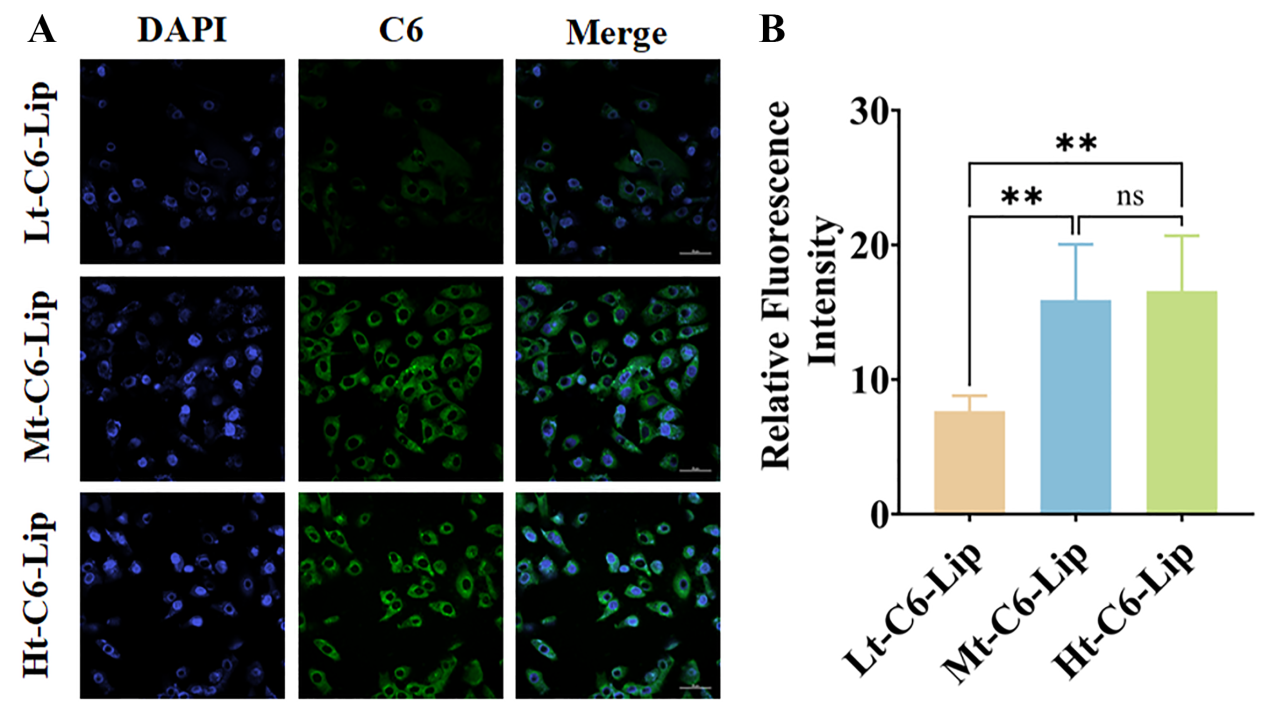


Figure S6. Optimisation of peptide A20FMDV2 loading on liposome.

(A) LSCM fluorescence images of Heap1-6 cell line after incubation with Free-C6, C6-Lip, and t-C6-Lip with different concentrations of peptide A20FMDV2 for 2 h;

(B) Semi-quantitative radiant efficiency analysis of the HCCC-9810 cell line uptake of t-C6-Lip with different concentrations of peptide A20FMDV2 (*n*=3). **, *p< 0.01*.

Note: Lt-C6-Lip: 5 mg/mL; Mt-C6-Lip: 10 mg/mL; Ht-C6-Lip: 20 mg/mL.

**4. HPLC and HPLC–MS/MS methods for the quantitative detection of Q7R**

Agilent 1260 HPLC was conducted to analyse the content of Q7R in liposome using a Thermo Fisher HyPURITY C18 column (4.6 mm × 150 mm, 3 μm); Acetonitrile/Water (80:20, *v/v*) as mobile phase at a flow rate of 1.0 mL/min with a run time of 15 minutes. Column temperature, 35 °C; UV detection wavelength, 375 nm; Injection volume: 10 μL, the representative HPLC chromatograms as shown in Figure S7. Assay validation including the evaluation of the calibration curve accuracy, accuracy, precision and stability were performed according to the previous studies. The correlation coefﬁcients of the calibration curves exceeded 0.999 [(Table S3](#_bookmark15)). The within-run accuracy varied between 97.61 to 107.66%, and the relative standard deviation (RSD%) of precision was below 5.48%. The between-run accuracy ranged from 96.33 to 104.10%, with the RSD% of precision lying below 5.97% (Table S4). The above data indicated that the developed method is reliable and offers good precision and accuracy. The all RSDs of repeatability lying below 5.00%, and obtained data showed that the Q7R remained stable for at least six months at -80 °C, 12 h at 4 °C and 25 °C, and three freeze–thaw cycles (Table S5).


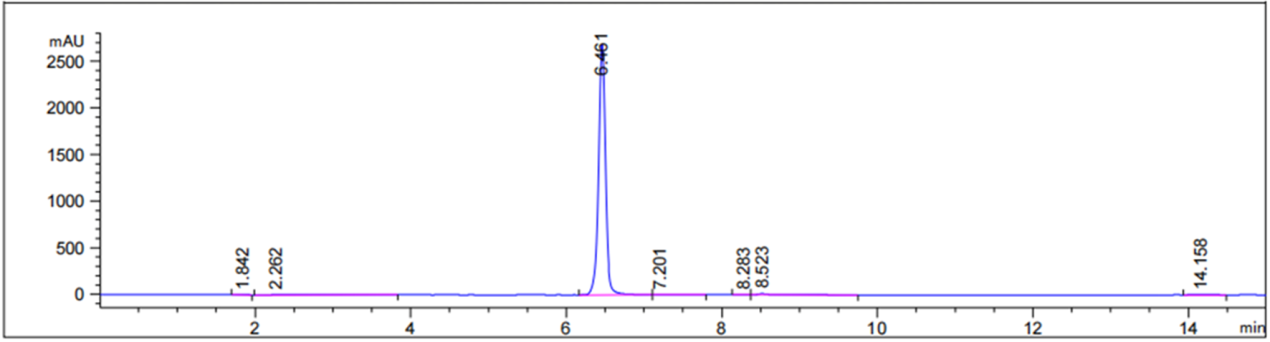


Figure S7. Representative HPLC chromatograms of Q7R.

Table S3. Calibration parameters of Q7R.

|  | Standard Curve | R² | Range (µg/mL) |
| --- | --- | --- | --- |
| Day 1 | *Y* = 136.18 *X* - 160.38 | 0.9994 | 0.78125-100 |
| Day 2 | *Y* = 140.28 *X* - 175.09 | 0.9992 |  |
| Day 3 | *Y*= 138.31 *X* - 133.73 | 0.9997 |  |

Table S4. Within-run and between-run precision and accuracy values of Q7R (*n*=3).

| Cpd. | Conc. (µg/mL) | Intra-day | | Inter-day | |
| --- | --- | --- | --- | --- | --- |
|  |  | Accuracy  (%) | Precision (%) | Accuracy (%) | Precision (%) |
| Q7R | 3.125 | 107.66±2.98 | 2.77 | 104.10±6.21 | 5.97 |
|  | 12.500 | 97.61±5.35 | 5.48 | 96.81±5.19 | 5.36 |
|  | 50.000 | 99.32±4.42 | 4.45 | 96.33±4.45 | 4.62 |

Table S5. Repeatability and Stability of Q7R under different conditions (*n*=3).

| Cpd. | Conc. (µg/mL) | Repeatability (RSD%) | Concentration (x±S.D.) | | | |
| --- | --- | --- | --- | --- | --- | --- |
|  |  |  | Autosampler (4 ºC) | Bench-top (25 ºC) | Freeze-thaw | Long-term  (-20 ºC) |
| Q7R | 3.125 | 2.87 | 3.42±0.11 | 3.23±0.27 | 3.18±0.15 | 3.15±0.19 |
|  | 12.500 | 2.43 | 12.45±0.42 | 12.26±0.45 | 11.88±0.43 | 11.45±0.14 |
|  | 50.000 | 1.64 | 51.05±2.13 | 46.49±0.62 | 47.41±1.69 | 50.03±1.47 |

Shimadzu LC-20AD tandem AB Sciex 3200 MD mass spectrometer with a Thermo Fisher HyPURITY C18 column (4.6 mm × 150 mm, 3 μm) was conducted to analyse the content of Q7R in *in vitro* release experiments. The mobile phase containing 0.1 % formic acid in water (Mobile phase A) and 0.1% formic acid in acetonitrile (Mobile phase B) was provided according to the following procedure: 0-5min: 20-55% B, 5-6min: 55-95% B, 6-10min: 95% B, 10-15min: 95-20% B, flow rate of 0.6 mL/min with a run time of 15 minutes. Column temperature, 35 °C; Injection volume: 10 μL. Tangeretin (Internal standard, TG): 373.1/343.2 (DP: 54.06, EP: 3.08, CE: 35.42, CXP: 5.23); Q7R: 449.1/303.1 (DP: 133.15, EP: 7.30, CE: 24.86, CXP: 4.45); Curtain Gas: 40 psi; IonSpray Voltage: 5500V; Ion Source Gas1: 55 psi; Ion Source Gas2: 60 psi; Interface Heater: On; Collision Gas: 6 psi, Temperature: 550 °C, the representative HPLC–MS/MS chromatograms as shown in Figure S8. Assay validation including the evaluation of the calibration curve accuracy, accuracy, precision and stability were performed according to the previous studies. The correlation coefﬁcients of the calibration curves exceeded 0.999 ([Table S6](#_bookmark15)). The within-run accuracy varied between 93.26 to 98.03%, and the RSD% of precision was below 7.42%. The between-run accuracy ranged from 95.58 to 97.69%, with the RSD % of precision lying below 6.73% (Table S7). The above data indicated that the developed method is reliable and offers good precision and accuracy. The mean recoveries of Q7R were between 88.28 and 105.61%, with all RSDs of repeatability lying below 10.00%. Furthermore, the obtained data showed that the Q7R remained stable for at least six months at -80 °C, 12 h at 4 °C and 25 °C, and three freeze–thaw cycles (Table S8).


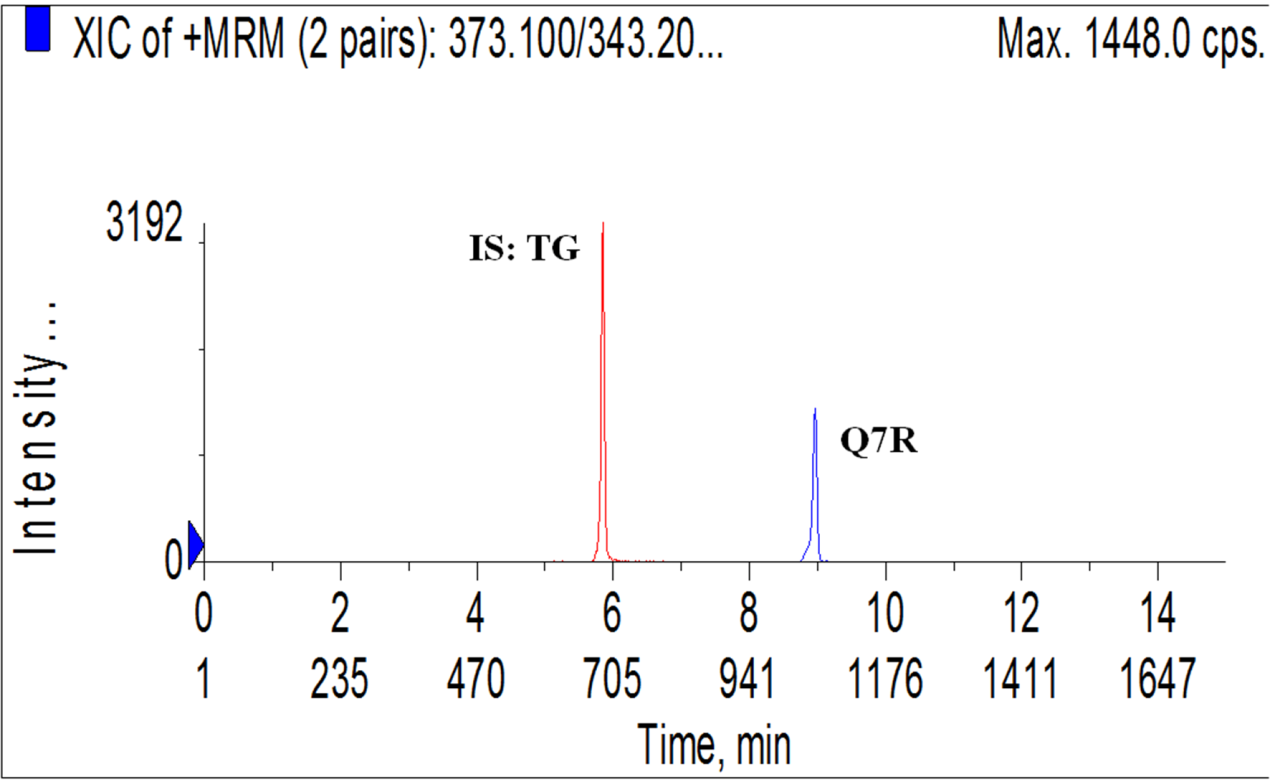


Figure S8. Representative HPLC–MS/MS chromatograms of Q7R.

Table S6. Calibration parameters of Q7R.

|  | Standard Curve | R² | Range (ng/mL) |
| --- | --- | --- | --- |
| Day 1 | *Y* = 0.0019 *X* + 0.015 | 0.9998 | 20-2000 |
| Day 2 | *Y* = 0.0017 *X* – 0.0056 | 0.9999 |  |
| Day 3 | *Y*= 0.0014 *X* + 0.0291 | 0.9993 |  |

Table S7. Within-run and between-run precision and accuracy values of Q7R (*n*=3).

| Cpd. | Conc. (ng/mL) | Intra-day | | Inter-day | |
| --- | --- | --- | --- | --- | --- |
|  |  | Accuracy  (%) | Precision (%) | Accuracy  (%) | Precision (%) |
| Q7R | 50.000 | 93.26±3.68 | 3.94 | 97.69±6.11 | 6.25 |
|  | 200.000 | 93.92±4.13 | 4.40 | 95.58±5.54 | 5.79 |
|  | 1000.000 | 98.03±7.27 | 7.42 | 96.41±6.49 | 6.73 |

Table S8. Repeatability and Stability of Q7R under different conditions (*n*=3).

| Cpd. | Conc. (ng/mL) | Repeatability (RSD%) | Concentration (x±S.D.) | | | |
| --- | --- | --- | --- | --- | --- | --- |
|  |  |  | Autosampler (4 ºC) | Bench-top (25 ºC) | Freeze-thaw | Long-term  (-20 ºC) |
| Q7R | 50.000 | 4.54 | 48.80±3.56 | 48.73±4.32 | 49.26±3.37 | 49.94±3.88 |
|  | 200.000 | 6.80 | 197.17±13.45 | 196.08±13.61 | 197.86±13.64 | 198.38±14.57 |
|  | 1000.000 | 6.10 | 939.21±62.92 | 950.74±85.90 | 958.57±56.41 | 944.92±50.75 |

**5. Hemolysis test**


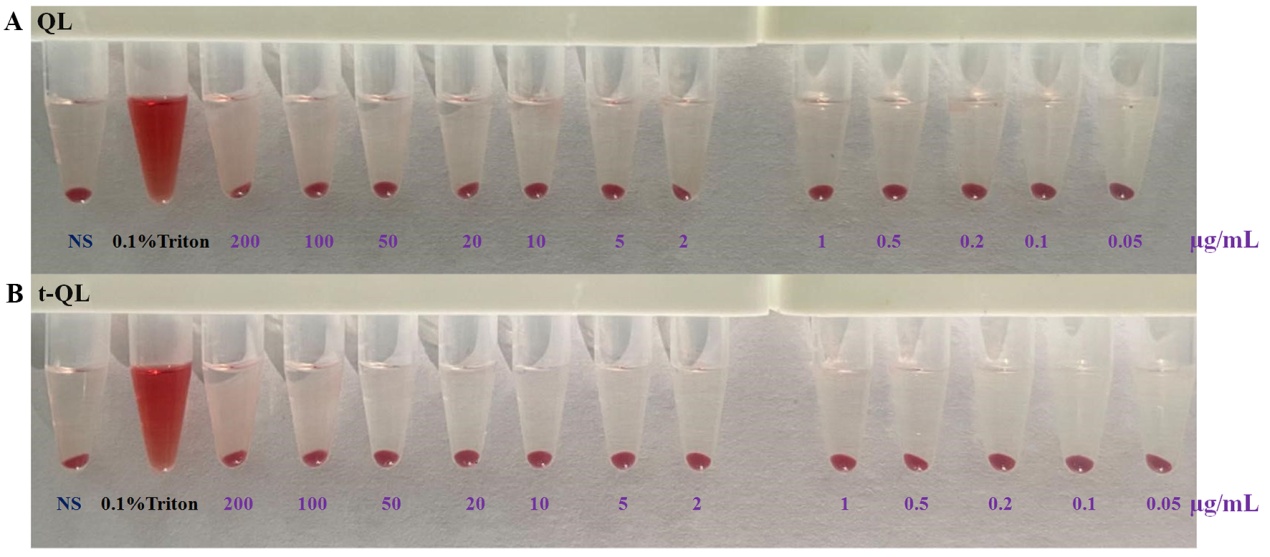


Figure S9. The visual inspection of QL and t-QL induced hemolysis.

**6. RT-PCR analysis**

Table S9. Primers used for RT-PCR

| Gene | Primer Sequence (5'-3') | Sequence length (bp) |
| --- | --- | --- |
| SIRT1 | Forward: AGATTTCAAGGCTGTTGGTTCC | 326 |
|  | Reverse: CAGCATCATCTTCCAAGCCATT |  |
| FXR | Forward: CCATTTACAAGCCACGGACG | 180 |
|  | Reverse: CCCAGGTTGGAATAATAGGACG |  |
| CYP27A1 | Forward: GAGACTGTCGGCACCTTTCCT | 179 |
|  | Reverse: ATGTGGGCAAAGTCCTTGTTCT |  |
| MFN2 | Forward: CTCCCTCTGACACCTGCCAA | 143 |
|  | Reverse: ACTCCTCCGACCACGAGAAT |  |
| BSEP | Forward: ACAAGATAAGATGAAGGAGGGAGAC | 102 |
|  | Reverse: CATACGCCTCCCATAAGCATC |  |
| CYP7A1 | Forward: GGCATCTCAAGCAAACACCAT | 246 |
|  | Reverse: GCTGTGCGGATATTCAAGGAT |  |
| ABCB4 | Forward: TCATCGCTGGGTTTGAGGA | 194 |
|  | Reverse: GCGTGCTTTCCAGCCATTT |  |
| PGC-1α | Forward: GAGAAGCGGGAGTCTGAAAGG | 219 |
|  | Reverse: GTCACAGGTGTAACGGTAGGTAATG |  |
| GAPDH | Forward: CTGGAGAAACCTGCCAAGTATG | 138 |
|  | Reverse: GGTGGAAGAATGGGAGTTGCT |  |

**7. Western blotting**

Table S10. The primary antibodies Used for Western blot analysis

| Primary Antibody | Company | Catalog No. | Dilution |
| --- | --- | --- | --- |
| CDS2 | Cohesion Biosciences | CQA4034 | 1:1000 |
| MFN2 | BIOSS | bs-2988R | 1:1000 |
| CYP27A1 | Affinity Biosciences | DF3571 | 1:1000 |
| FXR | Abcam | ab155124 | 1:1000 |
| GAPDH | Scrvicebio | GB11002 | 1:1000 |

**8. Serum bile acid profiles analysis by** **UPLC-MS/MS**

Serum (100 μL) was added to 500 μL of cold methanol, and 10 μL of 200 ng/mL mixed isotope internal standard solution. Vortexing for 1 min, and place in -20 °C for 1 hour to precipitate protein, the sample was centrifuged at 21,380 × g at 4 °C for 20 min, and the supernatant (500 µL) was collected and dried using a stream of dry N2 at 30 °C. The residue was re-dissolved in methanol (50 µL), and the solution was vortexed for 1 min and centrifuged at 21380 × g for 15 min at 4 °C. A 40 µL aliquot of the supernatant was collected for analysis.

The bile acid profiles were analyzed by Agilent 1290 Infinity UPLC tandem SCIEX 5500 QTRAP use an ACQUITY UPLC BEH C18 analytical column (1.7 μm, 2.1mm × 100mm) with negative ion model. The mobile phase containing 0.1 % formic acid in water (Mobile phase A) and methanol solution (Mobile phase B) was provided according to the following procedure: 0-7 minutes (50% -70% B); 7-15 minutes (70-90% B); 15-17 minutes (90% B); 17-17.1 minutes (90-60% B); 17.1-20 minutes (60% B). The injection volume and flow rate were 2 μL and 0.25 mL/min, column temperature was 45 °C. The following MS parameters were used: Source temperature: 550 °C; Ion Source Gas1 (Gas1): 55; Ion Source Gas2 (Gas2): 55; Curtain gas (CUR): 40; IonSapary Voltage Floating (ISVF): -4500 V. The multiple reaction monitoring parameters, and linear equation and range, LOD, LOQ, ULOQ were listed in Table S11. All of the UPLC-MS / MS data were obtained and analysed using Multiquant 3.0.2 softwar.

Table S11. The MRM parameter of UPLC-MS/MS detection and methodological investigation

| Metabolite Name | Mass Info | Linear | R | Linear range  (ng/mL) | LOD  (ng/mL) | LOQ  (ng/mL) | ULOQ  (ng/mL) |
| --- | --- | --- | --- | --- | --- | --- | --- |
| Allocholic acid (AlloCA) | 407.4 / 361.2 | *Y* = 0.00106 *X* - 0.01705 | r = 0.99908 | 0.5-2500 | 0.1 | 0.5 | 2500 |
| Apocholic acid (ApoCA) | 389.4 / 389.4 | *Y* = 0.00974 *X* - 0.57272 | r = 0.99983 | 0.5-2500 | 0.1 | 0.5 | 2500 |
| Chenodeoxycholic acid (CDCA) | 391.4 / 391.4 | *Y* = 0.00752 *X* + 0.33968 | r = 0.99913 | 0.5-2500 | 0.1 | 0.5 | 2500 |
| Cholic acid (CA) | 407.4 / 407.4 | *Y* = 0.00433 *X* + 0.15883 | r = 0.99786 | 0.5-2500 | 0.1 | 0.5 | 2500 |
| 3-Dehydrocholic acid (3-DHCA) | 405.4 / 405.4 | *Y* = 0.00881 *X* + 0.17964 | r = 0.99793 | 0.5-2500 | 0.1 | 0.5 | 2500 |
| Deoxycholic acid (DCA) | 391.4 / 391.4 | *Y* = 0.01172 *X* + 0.66877 | r = 0.99767 | 0.5-2500 | 0.1 | 0.5 | 2500 |
| 6,7-Diketolithocholic acid (CA) | 403.4 / 347.3 | *Y* = 0.00115 *X* - 0.11161 | r = 0.99837 | 0.5-2500 | 0.1 | 0.5 | 2500 |
| Glycochenodeoxycholic acid (GCDCA) | 448.4 / 74.0 | *Y* = 0.04267 *X* - 4.96446 | r = 0.99768 | 0.5-2500 | 0.1 | 0.5 | 2500 |
| Glycocholic acid (GCA) | 464.4 / 74.0 | *Y* = 0.00541 *X* - 0.15375 | r = 0.99994 | 0.5-2500 | 0.1 | 0.5 | 2500 |
| Glycodeoxycholic acid (GDCA) | 448.4 / 73.9 | *Y* = 0.00903 *X* - 0.08955 | r = 0.99864 | 0.5-2500 | 0.1 | 0.5 | 2500 |
| Glycohyodeoxycholic acid (GHDCA) | 448.4 / 74.1 | *Y* = 0.00807 *X* - 0.36111 | r = 0.99739 | 0.5-2500 | 0.1 | 0.5 | 2500 |
| Glycolithocholic acid (GLCA) | 432.5 / 73.9 | *Y* = 0.00682 *X* - 0.85617 | r = 0.99800 | 0.5-2500 | 0.1 | 0.5 | 2500 |
| Glycoursodeoxycholic acid (GUDCA) | 448.4 / 73.9 | *Y* = 0.00676 *X* - 0.29673 | r = 0.99934 | 0.5-2500 | 0.1 | 0.5 | 2500 |
| Hyodeoxycholic acid (HDCA) | 391.4 / 391.4 | *Y* = 0.00795 *X* - 0.17064 | r = 0.99786 | 0.5-2500 | 0.1 | 0.5 | 2500 |
| Isolithocholic acid (isoLCA) | 375.5 / 375.5 | *Y* = 0.00219 *X* + 0.03653 | r = 0.99988 | 0.5-2500 | 0.1 | 0.5 | 2500 |
| 7-Ketodeoxycholic acid (7-KDCA) | 405.4 / 405.4 | *Y* = 0.07438 *X* - 3.22024 | r = 0.99930 | 0.5-2500 | 0.1 | 0.5 | 2500 |
| 7-Ketolithocholic acid (7-KLCA) | 389.4 / 389.4 | *Y* = 0.00487 *X* - 0.16046 | r = 0.99904 | 0.5-2500 | 0.1 | 0.5 | 2500 |
| 12-Ketolithocholic acid (12-K LCA) | 389.4 / 389.4 | *Y* = 0.00249 *X* - 0.06331 | r = 0.99958 | 0.5-2500 | 0.1 | 0.5 | 2500 |
| Lithocholic acid (LCA) | 375.3 / 375.3 | *Y* = 0.00401 *X* - 0.06047 | r = 0.99818 | 0.5-2500 | 0.1 | 0.5 | 2500 |
| α-Muricholic acid (α-MCA) | 407.4 / 407.4 | *Y* = 0.01665 *X* + 0.00374 | r = 0.99949 | 0.5-2500 | 0.1 | 0.5 | 2500 |
| β-Muricholic acid (β-MCA) | 407.4 / 407.4 | *Y* = 0.01365 *X* - 0.02476 | r = 0.99946 | 0.5-2500 | 0.1 | 0.5 | 2500 |
| γ-Muricholic acid (γ-MCA) | 407.4 / 407.4 | *Y* = 0.01129 *X* + 0.05568 | r = 0.99776 | 0.5-2500 | 0.1 | 0.5 | 2500 |
| ω-Muricholic acid (ω-MCA) | 407.4 / 407.4 | *Y* = 0.00245 *X* + 0.03251 | r = 0.99790 | 0.5-2500 | 0.1 | 0.5 | 2500 |
| Tauro α-muricholic acid /Tauro β-muricholic acid/Tauro ω-muricholic acid (TMCA) | 514.4 / 79.8 | *Y* = 0.01275 *X* + 0.15910 | r = 0.99833 | 0.5-2500 | 0.1 | 0.5 | 2500 |
| Murideoxycholic Acid (CA) | 391.4 / 391.4 | *Y* = 0.00640 *X* + 0.08562 | r = 0.99781 | 0.5-2500 | 0.1 | 0.5 | 2500 |
| Taurochenodeoxycholic acid (TCDCA) | 498.4 / 80.0 | *Y* = 0.00519 *X* + 0.41637 | r = 0.99765 | 0.5-2500 | 0.1 | 0.5 | 2500 |
| Taurocholic acid (TCA) | 514.4 / 79.9 | *Y* = 0.00213 *X* + 0.07738 | r = 0.99854 | 0.5-2500 | 0.1 | 0.5 | 2500 |
| Taurodeoxycholic acid (TDCA) | 498.4 / 80.0 | *Y* = 0.01245 *X* + 0.00311 | r = 0.99738 | 0.5-2500 | 0.1 | 0.5 | 2500 |
| Taurohyocholic acid (THCA) | 514.4 / 79.8 | *Y* = 0.01275 *X* -0.19945 | r = 0.99735 | 0.5-2500 | 0.1 | 0.5 | 2500 |
| Taurohyodeoxycholic acid (THDCA) | 498.4 / 79.9 | *Y* = 0.00805 *X* + 0.14836 | r = 0.99844 | 0.5-2500 | 0.1 | 0.5 | 2500 |
| Taurolithocholic acid (TLCA) | 482.4 / 80.0 | *Y* = 0.00854 *X* - 0.01268 | r = 0.99980 | 0.5-2500 | 0.1 | 0.5 | 2500 |
| Tauroursodeoxycholic acid (TUDCA) | 498.4 / 79.8 | *Y* = 0.00803 *X* - 0.12883 | r = 0.99848 | 0.5-2500 | 0.1 | 0.5 | 2500 |
| Ursodeoxycholic acid (UDCA) | 391.4 / 391.4 | *Y* = 0.00706 *X* + 0.24563 | r = 0.99783 | 0.5-2500 | 0.1 | 0.5 | 2500 |


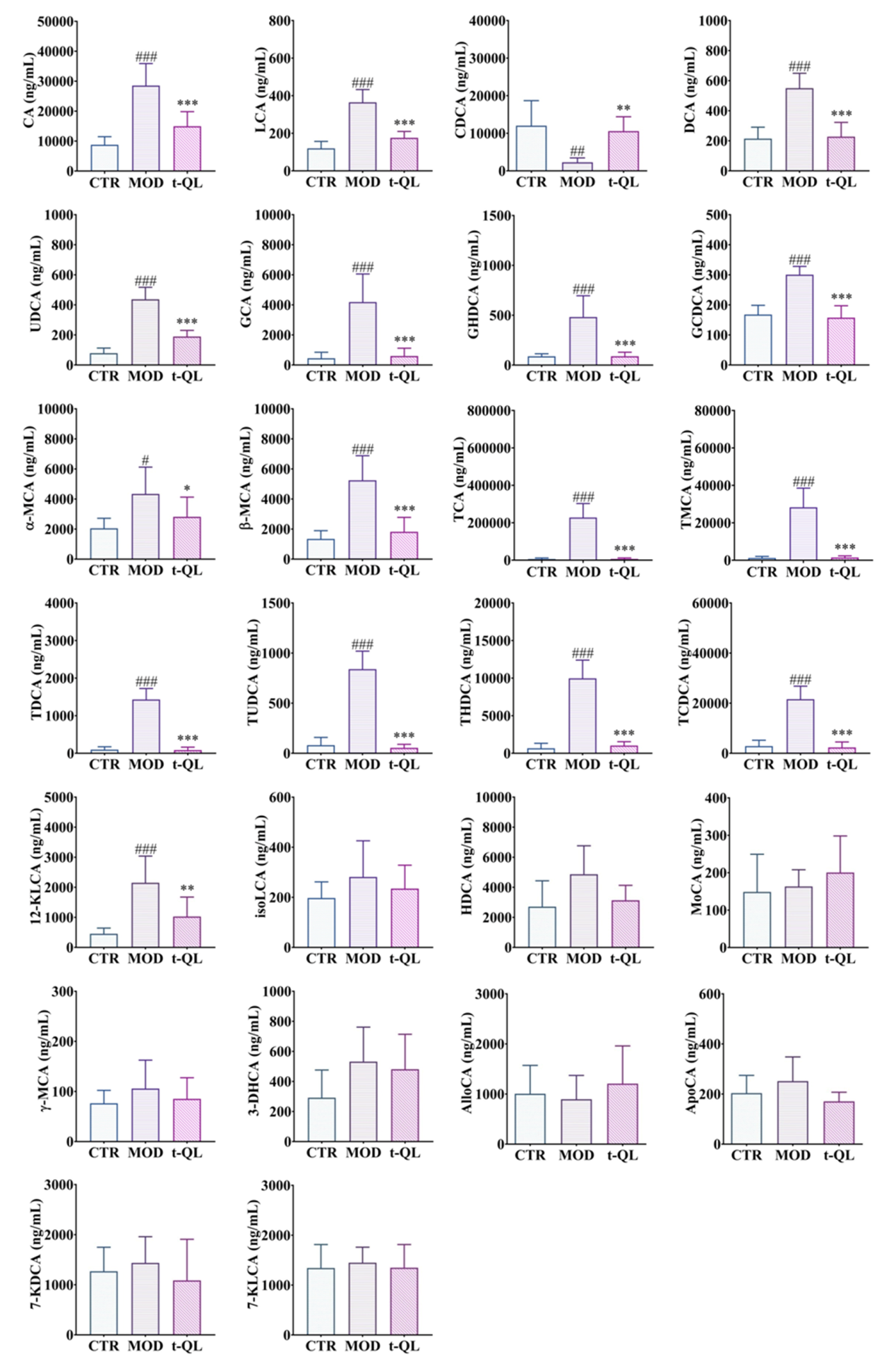


Figure S10. The level of BA metabolites (*n*=6). *, *p< 0.05*; **, *p< 0.01*; and ***, *p< 0.001* compared to the model group. ^#^, *p<0.05*; ^##^, *p<0.01;* ^###^, *p<0.001* compared to the control.


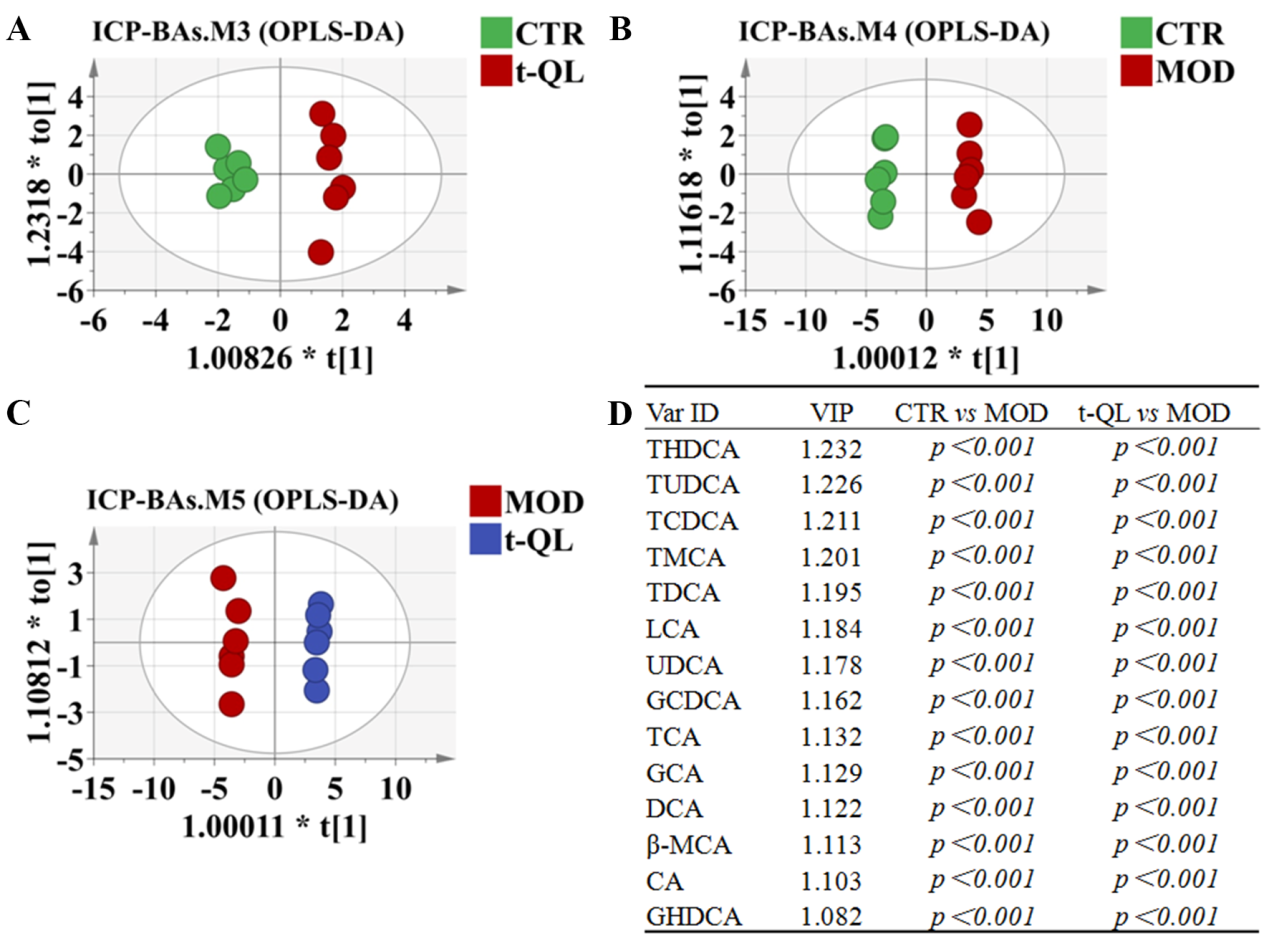


Figure S11. Analysis of potential bile acid biomarkers.

(A) OPLS-DA score plot of control and ICP model rat treated with t-QL, R2X(cum) = 0.358 and R2Y(cum) = 0.969, Q2(cum) = 0.120;

(B) OPLS-DA score plot of control and ICP model rat, R2X(cum) = 0.629 and R2Y(cum) = 0.993, Q2(cum) = 0.968;

(C) OPLS-DA score plot of ICP model rat untreated/treated with t-QL, R2X(cum) = 0.6261 and R2Y(cum) = 0.992, Q2(cum) = 0.971;

(D) Potential biomarkers of BA metabolites.

**
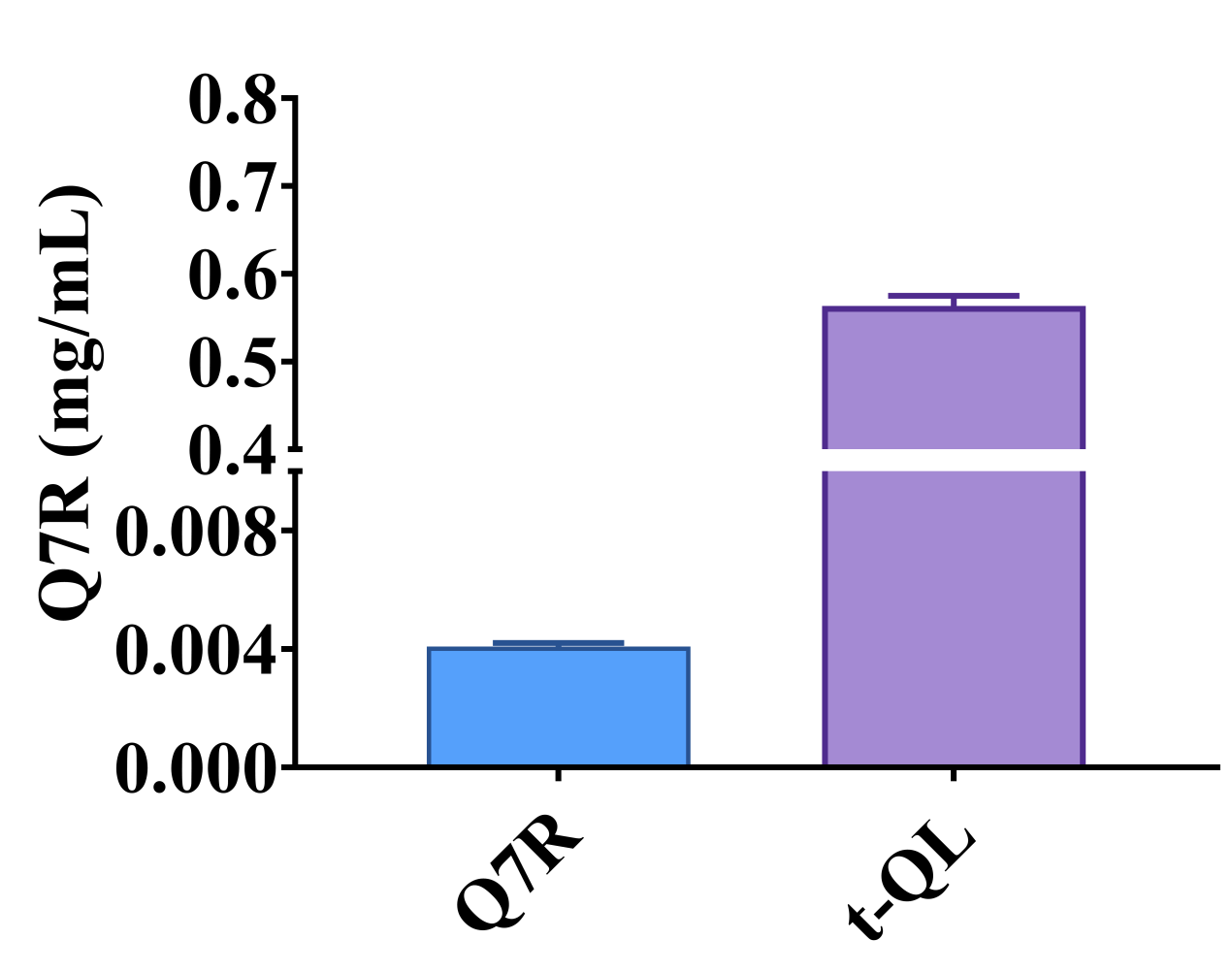
**

Figure S12. Solubility of Q7R and its concentration in liposomal solution
